# Pneumolysin Activates Macrophage Lysosomal Membrane Permeabilization and Executes Apoptosis by Distinct Mechanisms without Membrane Pore Formation

**DOI:** 10.1128/mBio.01710-14

**Published:** 2014-10-07

**Authors:** Martin A. Bewley, Michael Naughton, Julie Preston, Andrea Mitchell, Ashleigh Holmes, Helen M. Marriott, Robert C. Read, Timothy J. Mitchell, Moira K. B. Whyte, David H. Dockrell

**Affiliations:** ^a^ Department of Infection and Immunity, University of Sheffield Medical School, Sheffield, United Kingdom; ^b^ The Florey Institute for Host-Pathogen Interactions, University of Sheffield Medical School, Sheffield, United Kingdom; ^c^ Sheffield Teaching Hospitals NHS Trust, Sheffield, United Kingdom; ^d^ Institute of Microbiology and Infection, School of Immunity and Infection, University of Birmingham, Birmingham, United Kingdom; ^e^ James Hutton Institute, Dundee, United Kingdom; ^f^ Academic Unit of Clinical and Experimental Sciences and NIHR Respiratory Biomedical Research Unit, University of Southampton, Southampton, United Kingdom; ^g^ Department of Respiratory Medicine and MRC Centre for Inflammation Research, University of Edinburgh, Edinburgh, United Kingdom

## Abstract

Intracellular killing of *Streptococcus pneumoniae* is complemented by induction of macrophage apoptosis. Here, we show that the toxin pneumolysin (PLY) contributes both to lysosomal/phagolysosomal membrane permeabilization (LMP), an upstream event programing susceptibility to apoptosis, and to apoptosis execution via a mitochondrial pathway, through distinct mechanisms. PLY is necessary but not sufficient for the maximal induction of LMP and apoptosis. PLY’s ability to induce both LMP and apoptosis is independent of its ability to form cytolytic pores and requires only the first three domains of PLY. LMP involves TLR (Toll-like receptor) but not NLRP3/ASC (nucleotide-binding oligomerization domain [Nod]-like receptor family, pyrin domain-containing protein 3/apoptosis-associated speck-like protein containing a caspase recruitment domain) signaling and is part of a PLY-dependent but phagocytosis-independent host response that includes the production of cytokines, including interleukin-1 beta (IL-1β). LMP involves progressive and selective permeability to 40-kDa but not to 250-kDa fluorescein isothiocyanate (FITC)-labeled dextran, as PLY accumulates in the cytoplasm. In contrast, the PLY-dependent execution of apoptosis requires phagocytosis and is part of a host response to intracellular bacteria that also includes NO generation. In cells challenged with PLY-deficient bacteria, reconstitution of LMP using the lysomotrophic detergent LeuLeuOMe favored cell necrosis whereas PLY reconstituted apoptosis. The results suggest that PLY contributes to macrophage activation and cytokine production but also engages LMP. Following bacterial phagocytosis, PLY triggers apoptosis and prevents macrophage necrosis as a component of a broad-based antimicrobial strategy. This illustrates how a key virulence factor can become the focus of a multilayered and coordinated innate response by macrophages, optimizing pathogen clearance and limiting inflammation.

## IMPORTANCE

*Streptococcus pneumoniae*, the commonest cause of bacterial pneumonia, expresses the toxin pneumolysin, which can make holes in cell surfaces, causing tissue damage. Macrophages, resident immune cells essential for responses to bacteria in tissues, activate a program of cell suicide called apoptosis, maximizing bacterial clearance and limiting harmful inflammation. We examined pneumolysin’s role in activating this response. We demonstrate that pneumolysin did not directly form holes in cells to trigger apoptosis and show that pneumolysin has two distinct roles which require only part of the molecule. Pneumolysin and other bacterial factors released by bacteria that have not been eaten by macrophages activate macrophages to release inflammatory factors but also make the cell compartment containing ingested bacteria leaky. Once inside the cell, pneumolysin ensures that the bacteria activate macrophage apoptosis, rather than necrosis, enhancing bacterial killing and limiting inflammation. This dual response to pneumolysin is critical for an effective immune response to *S. pneumoniae*.

## INTRODUCTION

*Streptococcus pneumoniae*, the pneumococcus, is a significant cause of morbidity and mortality worldwide, contributing to an estimated 2 million deaths per annum. It is the most frequent cause of community-acquired pneumonia (1). The toxin pneumolysin (PLY) is a major virulence factor of *S. pneumoniae* and is expressed by almost all clinical isolates (2–4). PLY is a member of the cholesterol-dependent cytolysin (CDC) family of proteins expressed by a range of bacteria (5, 6). Monomeric soluble PLY binds to cholesterol-containing membranes and, through adjustment of its four domains, oligomerizes to form a ring-shaped pore (7), which can cause cytolysis in mammalian cells (8). PLY’s role in *S. pneumoniae* pathogenicity is well documented, with several studies showing that mice infected with PLY-deficient strains of *S. pneumoniae* display increased survival time (2), lower bacterial numbers in the nasopharynx (8), and decreased bacteremia and less lung damage (9, 10). PLY can also subvert complement-mediated opsonization (10–12). However, PLY also activates immune responses to pneumococci which contribute to host defense (13). PLY activates T cells (14), induces prostaglandin production in neutrophils (15), and promotes tumor necrosis factor alpha (TNF-α) production in mononuclear phagocytes (16). Pneumolysin is capable of activating inflammasomes, including members of the nucleotide-binding oligomerization domain (Nod)-like receptor family, pyrin domain-containing protein 3 (NLRP3), and absent in melanoma 2 (AIM 2), in both dendritic cells and macrophages. This leads to caspase 1 activation and secretion of interleukin-1 beta (IL-1β), a process which requires the cytolytic activity of the molecule and leads to protective immunity to *S. pneumoniae* (17, 18).

Macrophages are an essential contributor to the host response to pneumococcal infection, being responsible for the initial detection of bacteria and subsequent modulation of the inflammatory response (19). *S. pneumoniae*-infected macrophages undergo host-mediated programmed cell death (PCD), which mediates bactericidal activity in the later stages of the macrophage’s interaction when other killing mechanisms are exhausted and which is a prerequisite for bacterial clearance and resolution of inflammation (20–22). This form of PCD, which is significantly reduced during infections using PLY-deficient strains (21, 23), proceeds along a lysosome-mitochondrion axis involving initial lysosomal/phagolysosomal membrane permeabilization (LMP), which precedes the sequential loss of the inner mitochondrial transmembrane potential (Δψ_m_), mitochondrial outer membrane permeabilization (MOMP), caspase 3 activation, and, finally, nuclear condensation and fragmentation reminiscent of “classical” apoptosis (20). Moreover, the cell membrane remains intact until late in the cell death process and permeabilization is a prominent feature only when key regulators of the death program are inhibited (23). In this form of PCD, loss of lysosomal acidification (LLA) is a marker of LMP, which is an apical event in the initiation of PCD, linking activation of the lysosomal protease cathepsin D with regulation of cytosolic proteins that control the execution of the cell death program in the macrophage (20). PLY contributes to both LLA and cathepsin D activation (20). However, although PLY contributes to PCD, it is not sufficient, with internalization of live bacteria being required to unmask the role of PLY (23, 24). This contrasts with PLY-induced PCD in neurons (25, 26), cochlear hair cells (27), lymphocytes (28), and peritoneal macrophages (29) in which exogenous PLY alone can induce PCD directly and implies that host-mediated macrophage PCD in response to PLY proceeds through an alternative mechanism in differentiated macrophages.

In this study, we explored how PLY activates host-mediated macrophage PCD. We show that PLY contributes to PCD induction through two distinct processes that are independent of its pore-forming capacity. PLY enhances LMP, contributing to a phagocytosis-independent process involving Toll-like receptor (TLR) signaling, but is also required to ensure that, after bacterial internalization, LMP activates a form of PCD with features of apoptosis rather than a form of cell death with prominent plasma membrane permeabilization and cytolysis.

## RESULTS

### Pneumolysin’s pore-forming ability is not required for host-mediated macrophage apoptosis.

Cholesterol-dependent cytolysins (CDCs) exert many of their biological effects through pore formation (30). To examine the role of pore formation and of specific domains of PLY in induction of macrophage apoptosis, we studied a range of *S. pneumoniae* mutants expressing PLY. The mutant’s expression of PLY and ability to induce hemolysis were in keeping with existing knowledge of the protein’s function (see Fig. S1A and B in the supplemental material). All mutants were internalized to the same extent (see Fig. S1C).

**FIG 1:**
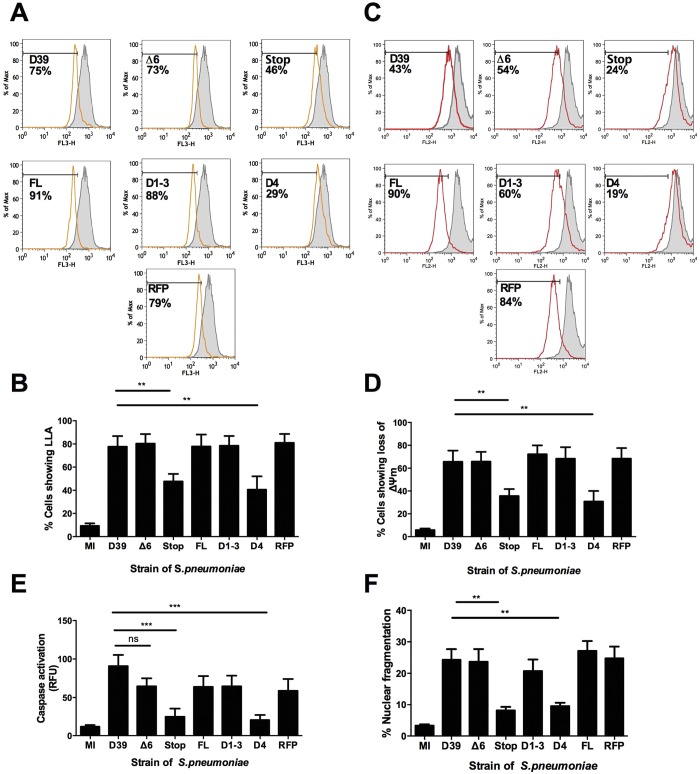
Pneumolysin’s pore-forming ability is not required for macrophage apoptosis. Monocyte-derived macrophages (MDM) were either mock infected (MI) or challenged with wild-type *S. pneumoniae* (D39), a D39 mutant expressing noncytolytic pneumolysin (Δ6), a pneumolysin-deficient D39 mutant (Stop), or reconstituted mutants expressing full-length pneumolysin (FL), pneumolysin domains 1 to 3 (D1-3), pneumolysin domain 4 only (D4), or red fluorescent protein-tagged pneumolysin (RFPPLY). (A to D) At 16 h postchallenge, cells were analyzed for loss of lysosomal acidification (LLA) (A and B) (*n* = 10) or for loss of the inner mitochondrial transmembrane potential (Δψm) (C and D) (*n* = 9). Representative histograms show the mock-infected sample in solid gray and the indicated mutant in white, with the percentage of cells showing loss of the marker indicated (A and C) and summary graphs of all data below (B and D). (E) At 16 h postchallenge, caspase 3 activity was measured (*n* = 8). (F) At 20 h postchallenge, cells were assessed for nuclear fragmentation (*n* = 9). In all experiments, ** = *P* < 0.01, *** = *P* < 0.001 (one-way ANOVA). All data are expressed as means ± standard errors of the means (SEM). Max, maximum; RFU, relative fluorescence units.

The ability of each mutant to induce apoptosis was examined at each step of the apoptotic pathway, commencing at LLA, as a marker of LMP, and progressing through sequential events in the apoptotic cascade (20). PLY was required for LLA, as the PLY-deficient strain, D39PLY-Stop (Stop), induced significantly lower levels of LLA than either the wild-type D39 strain or a strain complemented with full-length (FL) PLY (Fig. 1A and B). However, Δ6, a strain with no hemolytic activity, induced levels of LLA similar to those seen with strains D39 and FL as well as to those seen with a strain expressing red fluorescent protein (RFP)-tagged PLY. The same pattern was apparent for each of the subsequent stages of apoptosis that we examined: loss of Δψ_m_, caspase activation, and nuclear fragmentation (Fig. 1). To elucidate whether a particular region of PLY was required for LLA or apoptosis induction, macrophages were challenged with either *S. pneumoniae* strains expressing the first 3 domains (D1 to D3) or a strain expressing domain 4 only (D4), which is responsible for PLY’s membrane binding (31). Macrophages challenged with *S. pneumoniae* expressing D1 to D3 showed levels of each feature similar to those seen with D39-exposed cells, while exposure to strains expressing only D4 induced significantly lower levels (Fig. 1). As domains D1 to D3 are not hemolytic (31), these data corroborate the finding that the pore-forming ability of PLY is not required for apoptosis induction and suggest that the stimulus for LLA and apoptosis execution resides in D1 to D3.

### Pneumolysin mediates LLA and apoptosis through distinct pathways.

**FIG 2:**
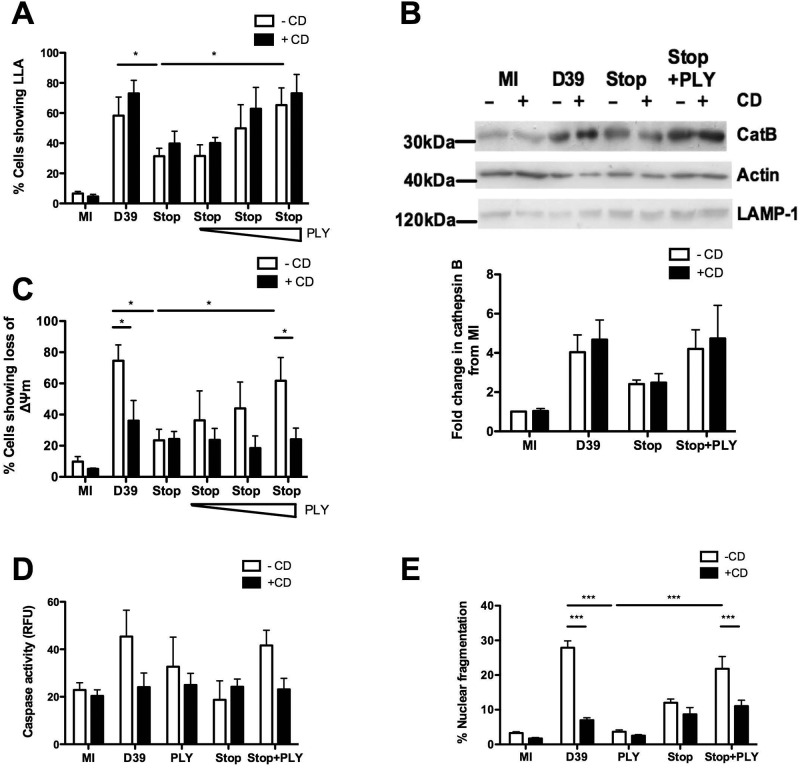
Pneumolysin mediates LLA and apoptosis through distinct pathways. Monocyte-derived macrophages (MDM) were mock infected (MI) or challenged with wild-type *S. pneumoniae* (D39), pneumolysin-deficient D39 (Stop), exogenous pneumolysin (PLY) alone, or Stop with exogenous pneumolysin (PLY), added at 0.5, 1, or 5 µg/ml (increasing doses are indicated a triangle) (A and C) or 5 µg/ml (all other panels) in the presence (+) or absence (-) of cytochalasin D (CD). At 16 h postchallenge, cells were assessed for loss of lysosomal acidification (LLA) (*n* = 4) (A), fractionated and the cytosolic fractions blotted for cathepsin B (Cat B), actin, or lysosome-associated membrane protein 1 (LAMP-1) (a representative blot from three independent experiments is shown, with densitometry data below the blot showing fold change in Cat B levels relative to MI cell levels) (*n* = 3) (B), assessed for loss of inner mitochondrial transmembrane potential (Δψm) (*n* = 4) (C), or assessed for caspase 3 activity (*n* = 4) (D), or cells were assessed for nuclear fragmentation at 20 h postchallenge (*n* = 5) (E). In all experiments, * = *P* < 0.05, ** = *P* < 0.01, *** = *P* < 0.001 (one-way or two-way ANOVA for comparisons within or between CD-negative and CD-positive [-CD and +CD] groups, respectively; data are expressed as means ± SEM). ns, not significant.

Since PLY directly induces cell death in a range of cell types and PLY is often a sufficient stimulus for PCD (13), we addressed whether the presence of PLY was sufficient to induce macrophage LLA and loss of Δψ_m_ as representative features of the apoptosis pathway of PCD observed in macrophages after exposure to *S. pneumoniae* (20). As shown in Fig. S2A and B in the supplemental material, exogenous PLY, added to reach a concentration of 5 µg/ml, failed to induce either LLA or loss of Δψ_m_. In the presence of bacteria, PLY was required for maximal apoptosis induction and exogenous PLY reconstituted levels of LLA and LMP and downstream features of the apoptosis pathway of PCD, observed in macrophages challenged with *S. pneumoniae*, when added to macrophages exposed to the PLY-deficient bacteria (Fig. 2). This indicates that PLY triggers LLA, LMP, and the apoptotic program only when combined with one or more additional microbial signals. In line with our prior observations, LLA was associated with LMP during pneumococcal challenge (Fig. 2A and B) and we found that PLY alone was also insufficient to activate cathepsin D (see Fig. S2C), an upstream effector of apoptosis activated in macrophage phagolysosomes during *S. pneumoniae*-associated PCD (20).

Macrophage PCD in response to *S. pneumoniae* is dependent upon internalization of bacteria (21). To address whether internalization of bacteria is also required for LLA or LMP, macrophages were treated with cytochalasin D, which inhibits actin polymerization (32) and phagocytosis of opsonized *S. pneumoniae* (21). Surprisingly, cytochalasin D did not alter LLA or LMP in macrophages challenged with *S. pneumoniae* compared to macrophages exposed to bacteria without cytochalasin D (Fig. 2A and B). PLY also reconstituted LLA or LMP in macrophages exposed to PLY-deficient bacteria and cytochalasin D (Fig. 2A and B). In contrast, cytochalasin D treatment inhibited all steps after LMP in the PCD pathway, including cathepsin D activation (see Fig. S2C in the supplemental material), loss of Δψ_m_ (Fig. 2C), caspase 3/caspase 7 activation (Fig. 2D), and nuclear fragmentation (Fig. 2E), indicating that all the steps involved in the execution of apoptosis required bacterial phagocytosis. To confirm that PCD resembled apoptosis, we also documented mitochondrial outer membrane permeabilization (MOMP) and caspase 9 activation. Furthermore, caspase 1 activity did not contribute to the death pathway and plasma membrane integrity remained intact until late in PCD (see Fig. S3).

### Recognition of cholesterol-dependent cytolysins and recognition of other microbial factors combine to induce loss of lysosomal acidification.

**FIG 3:**
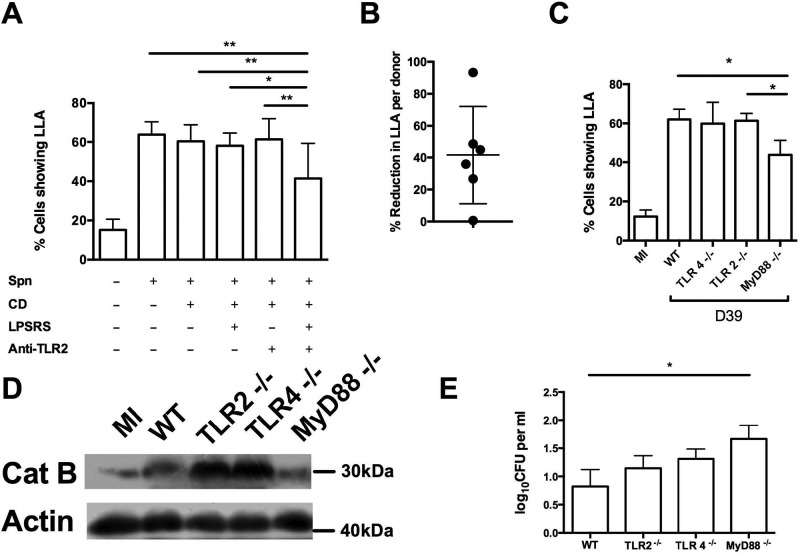
Toll-like receptor 2 (TLR2) signaling and TLR4 signaling combine to induce loss of lysosomal acidification. (A and B) Monocyte-derived macrophages (MDM) were challenged in the absence (-) or presence (+) of *S. pneumoniae* D39 (Spn), cytochalasin D (CD), a TLR4 antagonist (LPSRS), or a TLR2-blocking antibody (Anti-TLR2). At 16 h postchallenge, cells were analyzed for loss of lysosomal acidification (LLA) by flow cytometry (*n* = 6). * = *P* < 0.05, ** = *P* < 0.01 (one-way ANOVA). (B) The percentages of reduction in LLA after D39 challenge for individual donors, comparing untreated cells and those treated with both anti-TLR2 and LPSRS in the absence of CD. (C to E) Bone marrow-derived macrophages (BMDM) from wild-type (WT) or Toll-like receptor 2 (TLR2)-, TLR4-, or myeloid differentiation factor 88 (MyD88)-deficient mice were challenged with strain D39. At 16 h postchallenge, cells were analyzed for loss of lysosomal acidification (LLA) (C) and lysosomal membrane permeabilization, as characterized by cathepsin B (Cat B) translocation to the cytosol (D). (E) At 8 h postchallenge, early bacterial killing was determined by a gentamicin protection assay. In all experiments, *n* = 4. * = *P* < 0.05 (one-way ANOVA). Data are expressed as means ± SEM.

As the response to PLY that led to LLA and LMP required additional stimuli from whole bacteria but did not require phagocytosis, we tested whether 2 cell surface pattern recognition receptors (PRRs) potentially contributing to the host response to *S. pneumoniae* in macrophages contributed to LLA/LMP (33). Toll-like receptor 2 (TLR2) is known to play a role in the early inflammatory response to *S. pneumoniae* (34), and, although TLR4’s roles are debated, TLR4 appears to contribute to PLY recognition in macrophages (35) and has been implicated in apoptosis induction (29). To maximize the extracellular signal, these experiments were also performed in the presence of cytochalasin D. Although blockade of TLR2 or TLR4 abrogated lipopolysaccharide (LPS)- and lipotochoic acid (LTA)-mediated cytokine production (see methods), blockade of either TLR2 or TLR4 alone did not prevent LLA in D39-challenged cells. However, the combination of the two partially inhibited LLA (Fig. 3A) to an extent that varied with the donor (Fig. 3B). Bone marrow-derived macrophages (BMDM) lacking the MyD88 adaptor protein that regulates one of the TLR signaling pathways and a pathway utilized by both TLR2 and TLR4 also demonstrated a reduction in LLA or LMP (Fig. 3C and D), while those lacking TLR2 or TLR4 individually did not. This suggested that signaling through both TLR2 and TLR4, and potentially other PRRs, stimulated LLA or LMP. In keeping with the redundancy associated with TLR responses to pneumococci, only MyD88^−/−^ BMDM had a reduction in early bacterial killing (Fig. 3E). To assess if there was a contribution to LLA from the intracellular PRR nuclear oligomerization domain protein 2 (Nod2), a known sensor of internalized *S. pneumoniae* peptidoglycan (36), which may be engaged by microbial factors internalized through pinocytosis or other pathways not inhibited by cytochalasin D, cells were incubated with agonists of Nod 1 and/or Nod 2. However, these failed to induce LLA (see Fig. S4A in the supplemental material).

To assess if LLA was strain or species specific, we assessed LLA in cells challenged with clinical strains of serotype 1 *S. pneumoniae* expressing hemolytic (strain ST227) (37) or nonhemolytic (strain ST306) PLY (38) or in cells challenged with *Streptococcus mitis*, which expresses a cytolysin related to PLY, mitilysin (39). These strains all induced LLA and downstream features of PCD at levels comparable to those seen with D39 (see Fig. S4B to F in the supplemental material). Thus, a conserved response to PLY and related cytolysins, acting in concert with one or more additional microbial factors, induces LLA in a phagocytosis-independent fashion that also requires PRR activation.

### Cytoplasmic translocation of pneumolysin is associated with a progressive increase in lysosomal membrane permeabilization.

**FIG 4:**
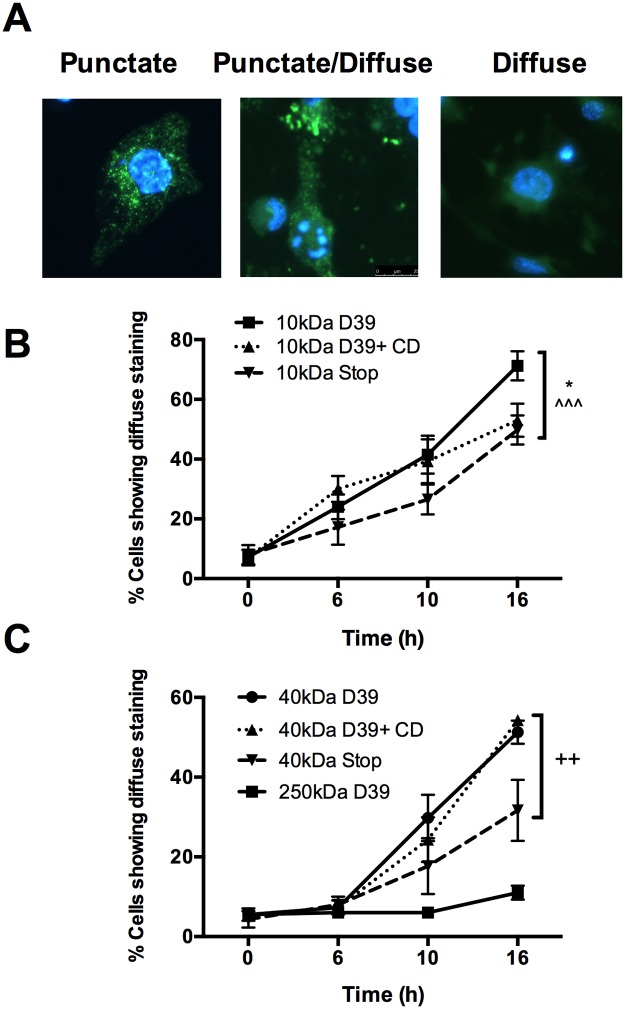
Macrophages exposed to *S. pneumoniae* develop a progressive increase in lysosomal membrane permeabilization. (A to C) Monocyte-derived macrophages (MDM) were preloaded with 5 mg/ml of FITC-dextran molecules of the designated molecular mass and challenged with *S. pneumoniae* (D39) or pneumolysin-deficient (Stop) *S. pneumoniae* or with D39 in the presence of cytochalasin D (D39+CD). At the designated time postchallenge, cells were fixed and analyzed by fluorescence microscopy and the percentages of cells showing mixed punctate/diffuse or diffuse staining were determined. (A) Representative images of cells showing completely punctate FITC-dextran localization, mixed punctate/diffuse staining, or completely diffuse staining, taken from a strain D39-exposed population at 10 h postchallenge. (B and C) Localization of 10-kDa (B) and 40- or 250-kDa (C) FITC-dextran (*n* = 4). * = *P* < 0.05 for 10-kDa D39 versus 10-kDa D39+CD; ^ ^ ^ = *P* < 0.001 for 10-kDa D39 versus 10-kDa Stop; ++ = *P* < 0.01 for 40-kDa D39 versus 40-kDa Stop (two-way ANOVA). Data are expressed as means ± SEM.

The requirement for intracellular bacteria for the execution phase of macrophage apoptosis implicates intracellular microbial factors, but we know little concerning the extent of LMP and its relationship to the cellular localization of PLY after the initial trafficking of bacteria into the phagolysosome (40). Macrophages that have internalized pneumococci exhibit significant LLA from 10 h postinfection and show evidence of LMP as determined by translocation of lysosomal proteases into the cytoplasm (20). However, lysosomal integrity could theoretically be compromised earlier to a more modest extent, allowing efflux of small peptides before LLA and cathepsin release can be detected. To determine more accurately the kinetics and extent of lysosomal and phagolysosomal permeabilization, the translocation of fluorescein isothiocyanate (FITC)-dextran molecules of various sizes, represented by the replacement of the normal punctate staining pattern by a mixed punctate/diffuse or diffuse pattern (Fig. 4A), was measured. There was progressive translocation of 10-kDa FITC-dextran molecules into the cytosol, with the translocation visible 6 h after bacterial challenge (Fig. 4B). The redistribution of the 40-kDa FITC-dextran molecules occurred by the 10-h time point (Fig. 4C). In the cases of both the 10-kDa and the 40-kDa molecules, translocation predominantly resulted in a mixture of both diffuse staining and punctate staining, indicative of partial lysosomal/phagolysosomal efflux. In contrast, the 250-kDa FITC-dextran remained in the lysosomal/phagolysosomal compartment at all time points (Fig. 4C).

In line with our observations with LLA, treatment of macrophages with cytochalasin D had little effect on efflux of 10-kDa or 40-kDa molecules (Fig. 4B and C). As documented with LLA, challenge with PLY-deficient bacteria partially reduced lysosomal leakage of 10-kDa and 40-kDa FITC-dextran, suggesting that PLY, along with additional microbial factors, contributed to LMP. These data corroborate our prior observations that LLA is a marker of LMP (20). They also indicate that LMP is selective and that permeability to medium-sized particles increases progressively with time; permeability to small-sized molecules precedes LLA or permeability for medium-sized molecules, while lysosomes/phagolysosomes remain impermeable to larger particles. They also confirm that LMP for small-sized and medium-sized particles is not dependent on phagocytosis.

**FIG 5:**
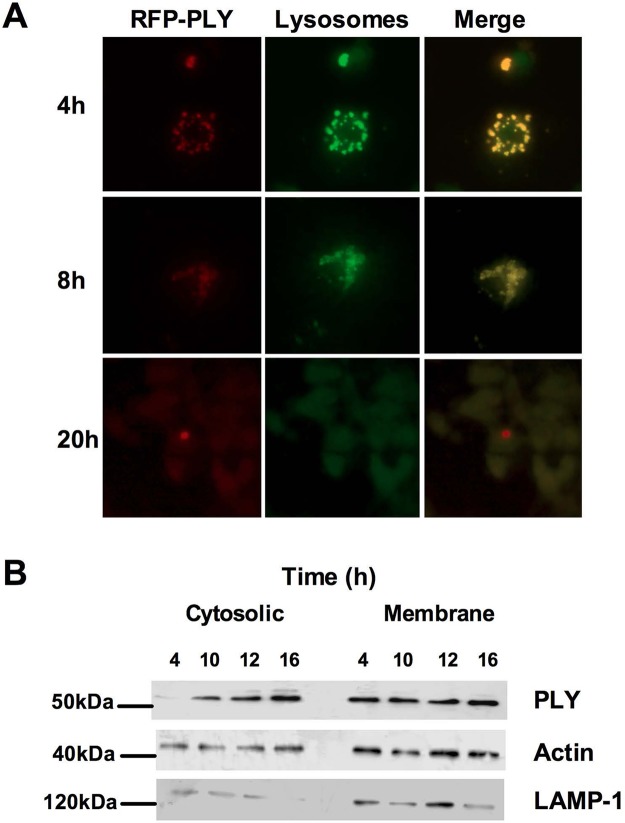
Pneumolysin progressively translocates from phagolysosomes to the cytosol following challenge with live bacteria. (A) Monocyte-derived macrophages (MDM) were challenged with *S. pneumoniae* (D39) expressing RFP-tagged pneumolysin (RFP-PLY). At the designated time postchallenge, cells were stained with BODIPY-pepstatin A to visualize lysosomes. Cells were visualized by fluorescence microscopy. Red, PLY; green, lysosomes; yellow, merged. Images are representative of the results of three independent experiments. (B) A Western blot of cytosolic and membrane fractions from D39-exposed MDMs at the designated time points postchallenge probed with anti-pneumolysin (PLY). Actin and lysosome-associated membrane protein 1 (LAMP-1) were used as loading controls. The blots are representative of the results of three independent experiments.

We next investigated the kinetics of PLY localization in relation to LMP, utilizing a mutant expressing full-length PLY with a red-fluorescent protein (RFP) label (RFP-PLY). At 4 h after exposure, red fluorescence could be seen in a punctate distribution which colocalized with the lysosomal marker BODIPY-pepstatin A (Fig. 5A). At 8 h postchallenge, the punctate distribution was still in evidence, but cells also showed a more diffuse red background staining, indicating that RFP-PLY was translocating to the cytosol. At 20 h postchallenge, virtually all red fluorescence was diffuse (Fig. 5A). Subcellular fractionation and Western blotting corroborated these findings, with evidence of significant cytosolic translocation of RFP-PLY by 10 h (Fig. 5B), suggesting that PLY translocation kinetics matched those of LMP for medium-sized particles.

### NLRP3 and ASC do not contribute to host-mediated macrophage apoptosis.

PLY’s translocation from the phagolysosome could activate a cytosolic pattern recognition receptor to stimulate the apoptosis pathway. PLY has been shown to activate both the cytosolic NLRP3 inflammasome (17) and the AIM 2 inflammasome, both of which recruit apoptosis-associated speck-like protein containing a caspase recruitment domain (ASC) (18). However, upon challenge with *S. pneumoniae*, murine NLRP3^−/−^ and ASC^−/−^ BMDM displayed percentages of cells with LLA (see Fig. S5A in the supplemental material), loss of Δψ_m_ (see Fig. S5B), and morphological nuclear features of apoptosis (see Fig. S5C) similar to those seen with control BMDM, suggesting that the pathways responsible for LLA or the downstream execution phase of apoptosis do not involve the NLRP3 or other ASC-containing inflammasomes. However, LLA, Δψ_m_, and nuclear fragmentation were significantly reduced when cells were challenged with the PLY-deficient Stop strain (Fig. 4). Caspase 1, which is activated via the inflammasome, also played no role in macrophage apoptosis in a murine model of lung infection (see Fig. S5D).

### Pneumolysin has distinct consequences for the macrophage innate immune response that differ with respect to their dependence on phagocytosis.

We next examined whether phagocytosis-independent and phagocytosis-dependent phases of the apoptosis response mapped to particular aspects of the macrophage’s PLY-mediated innate immune response to *S. pneumoniae* and the relationship of these immune responses to phagocytosis. As shown in Fig. S6A to D in the supplemental material, production of several cytokines was enhanced at later time points by PLY but was not phagocytosis dependent. This was particularly true for IL-1β and IL-6, with a similar trend seen for IL-8. In contrast, although we found little PLY dependence or influence on phagocytosis or production of reactive oxygen species (ROS) (see Fig. S6E and F), nitric oxide (NO) production was dependent both on PLY and on phagocytosis (see Fig. S6G). This positioned the phagocytosis-independent PLY response that contributed to LMP alongside effector functions associated with macrophage activation and cytokine release, but, in contrast, the phagocytosis-dependent PLY responses associated with apoptosis induction occurred in the context of macrophages that were activating delayed antimicrobial responses, including NO generation.

### Intracellular pneumolysin is necessary for optimal engagement of macrophage apoptosis.

**FIG 6:**
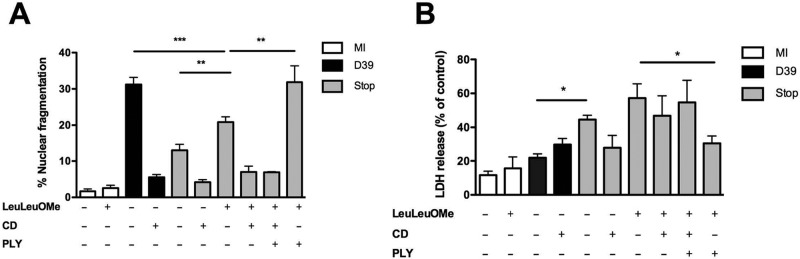
Pneumolysin contributes to maximal engagement of apoptosis. Monocyte-derived macrophages (MDM) were mock infected (MI; white bar) or challenged with either wild-type *S. pneumoniae* (D39; black bar) or pneumolysin-deficient D39 (Stop; gray bar). Cells were challenged in the presence (+) or absence (-) of the lysomotrophic detergent LeuLeuOMe, cytochalasin D (CD), or exogenous pneumolysin (5 µg/ml). At 20 h postchallenge, cells were assessed for nuclear fragmentation (*n* = 6) (A) or for necrosis (*n* = 5) (B) by measuring LDH release. * = *P* < 0.05, ** = *P* < 0.01, *** = *P* < 0.001 (one-way ANOVA). All data are expressed as means ± SEM.

PLY contributed to LMP independently of phagocytosis. In contrast, the execution phase of macrophage apoptosis required phagocytosis of bacteria, suggesting a role for intracellular microbial factors. Since PLY translocated to the cytosol, we addressed whether intracellular PLY was also required to execute apoptosis or was required solely to induce LMP. We reconstituted comparable levels of LMP in macrophages containing the PLY-deficient strain and determined whether apoptosis occurred to an extent similar to that observed with wild-type bacteria. The lysomotrophic detergent l-Leucyl-l-leucine methyl ester (LeuLeuOMe) is a dipeptide which, in lysosomes, is converted by the lysosomal enzyme dipeptidyl peptidase into a detergent-like compound which induces LMP (41, 42). The dose of LeuLeuOMe was selected as the dose that provided levels of LLA and kinetics comparable to those observed following pneumococcal challenge (see Fig. S7A in the supplemental material). It was also confirmed that it reconstituted LLA and LMP in cells challenged with Stop to an extent comparable to that observed in D39-exposed cells (see Fig. S7B and C). The lysomotrophic detergent did not induce significant apoptosis in the absence of bacteria but enhanced apoptosis following phagocytosis of PLY-deficient bacteria, though not to the extent seen with D39 bacteria (see Fig. 6A; see also Fig. S8A). In contrast, addition of PLY to cells exposed to the lysomotrophic detergent in the presence of Stop increased levels of apoptosis over those seen in the absence of PLY (see Fig. 6A; see also Fig. S8A), whereas PLY had no additional effect on LMP (see Fig. S7C). This that suggested PLY contributed to induction of apoptosis in this system downstream of its induction of LMP.

To establish the fate of cells in the absence of PLY-induced stimulus for apoptosis induction, we examined rates of overall cell death using lactate dehydrogenase (LDH) release as a marker of cell membrane permeabilization. When we examined LDH release in the same experiments in which we reconstituted LMP with lysomotrophic detergents, we observed that the combination of LMP and phagocytosed intracellular bacteria in the absence of PLY resulted in increased levels of LDH and presumably nonapoptotic cell death (Fig. 6B). The enhanced LDH release in the absence of PLY occurred irrespective of the presence or absence of LeuLeuOMe treatment and was not modified by the necroptosis inhibitor necrostatin. There was also no evidence of increased receptor-interacting serine/threonine protein kinase 1 (RIP1) or RIP3 levels indicative of necroptosis (43, 44) (see Fig. S8B and C in the supplemental material), suggesting that, in the absence of PLY, the macrophages died by toxin-induced cytolysis rather than through active engagement of a program of necroptosis. Similarly, we found higher rates of LDH release when macrophages were exposed to bacteria that expressed only D4 and therefore did not engage apoptosis as effectively, while bacteria expressing D1 to D3 or Δ6 bacteria induced levels comparable to those seen with wild-type bacteria (see Fig. S8C and D). This emphasizes the role of PLY in ensuring that apoptotic cell death is executed in preference to other forms of PCD during exposure to *S. pneumoniae.*

## DISCUSSION

Macrophage PCD during *S. pneumoniae* infection is a host-mediated response which is essential for the later stages of bacterial killing and pulmonary clearance of bacteria (22, 45). LLA and LMP are an apical part of the PCD engaged in macrophages following ingestion of *S. pneumoniae*. Here we show that PLY contributes to both LMP and the subsequent execution of PCD through distinct mechanisms. These act in concert to trigger the apoptotic demise of the macrophage. We show that PLY’s contribution to apoptosis does not involve PLY pore formation but requires phagocytosis-independent PRR-generated signals, which prime LMP and also complementation of LMP-derived signals to ensure execution of apoptosis.

PLY forms pores in cholesterol-containing membranes as a major mechanism underpinning its biological activity (13, 46). Neurons, microglia, and dendritic cells appear sensitive to low concentrations of PLY, and pore formation triggers PCD (25, 47), while fibroblasts and alveolar epithelial cells are less susceptible *in vitro* (48, 49). PLY pore formation triggers LMP in dendritic cells (17). PLY-induced pores also contribute to lymphocyte apoptosis (50), while exogenous PLY is a sufficient stimulus to induce apoptosis for relatively undifferentiated phenotypes of murine macrophages (29). We found no evidence that pore-forming activity was required for LMP or apoptosis in differentiated macrophages. Compositional differences in the plasma membrane and altered levels of PLY inactivators such as reactive oxygen species (ROS) result in various levels of susceptibility of cell membranes to PLY-induced pores and are potential contributors to the relative levels of resistance of differentiated macrophages to PLY pore-mediated effects (51).

PLY played two distinct roles in the induction of PCD. The first was phagocytosis independent and resulted in LMP. This response was potentially extracellular or could have involved PLY or other LMP-triggering ligands entering the cell through pinocytosis or other actin-independent mechanisms. LMP was stimulated under conditions similar to those associated with the production of several cytokines, including IL-1β. This observation suggested that PRR, involved in initial macrophage activation and priming of host defense by *S. pneumoniae*, could be involved in LMP induction (29, 34, 35, 52), and we confirmed potential roles for MyD88 and combined TLR2 and TLR4 signaling in generating LMP. TLR4 has been suggested to contribute to recognition of PLY in peritoneal macrophages, leading to cytokine production (35). However, the involvement of TLR4 in PLY recognition has been debated. Its relative importance in PLY recognition may vary with cell type, and different PLY-activated signaling pathways may vary in their requirements for TLR4 recognition. For example, while NF-κB signaling in macrophages in response to PLY was TLR4 dependent (35), nuclear factor of activated T cells (NFAT) (53) and p38 mitogen-activated protein kinase (MAPK) signaling in epithelial cells was TLR4 independent (54). Our results are consistent with prior findings linking TLR4-mediated PLY responses to induction of murine macrophage apoptosis (25, 29) but suggest that it is the integration of signals from multiple PRRs that generates LMP for differentiated human macrophages, although the relative contributions of TLR differed between donors. This may be representative of macrophage heterogeneity in levels of TLR expression, which occurs as macrophages differentiate and which is thought to add diversity to the immune response and may have differed among donors (55). The region of the toxin responsible for this interaction lies within domains 1 to 3 of PLY. The fact that a range of clinical strains and the related oral commensal *S. mitis* all induced LMP suggests that the recognition of the cytolysin and LMP induction are conserved and not restricted to just *S. pneumoniae*. The association of macrophage activation for cytokine release with LMP fits with observations that LMP facilitates inflammasome activation and optimal release of IL-1β (17). However, there was a role for TLR activation in priming LMP and early bacterial killing in the differentiated macrophage, while LMP did not require PLY pores. Therefore, TLR activation emerges as a mechanism of LMP that functions as an alternative to the direct induction by cytolysin-induced pore formation and appears to be the preferred mechanism operating in differentiated macrophages. These findings are most consistent with models where both initial TLR signals and subsequent NLR signals combine to optimize release of IL-1β and potentially other cytokines as well as mediating bacterial killing cascades (56, 57). Host-mediated LMP would enhance this response.

The specific signaling that led to LMP remains poorly defined. Phagocytosis-linked changes in kinase signaling or lipid composition are inconsistent with the lack of effect of cytochalasin D treatment (58). LMP precedes activation of caspase and other apoptosis-associated proteases that activate LMP in other settings in this form of PCD (20). The most plausible stimuli would appear to be those arising from PRR activation: mitogen-activated protein kinase (MAPK) signaling, enzymes regulating lipid membrane composition, such as phospholipase A2, and ROS-induced peroxidation of lipid membranes (33, 58, 59). To our knowledge, this is the first reported instance of a toxin recognition system triggering LMP to engage a host response. In contrast, toxins permeabilizing endosomes, as exemplified by diphtheria toxin, are well described (60). Peptides derived from PLY and potentially other microbial factors could translocate to the cytosol early after bacterial phagocytosis and interact with host proteins, enhancing cytokine release and providing a feedback loop to allow LMP for larger molecules, including PLY. Our data, however, exclude a role for the intracellular Nod2, NALP3, or other ASC-containing pneumococcal recognition systems in LMP or apoptosis induction.

Although PLY and other pore-forming bacterial toxins (e.g., listeriolysin O, streptolysin O, and α-hemolysin) activate the NLRP3 inflammasome (61–64), which activates caspase 1 (17), pneumococci limit caspase 1 activation in macrophages through induction of chitinase 3-like protein 1 (65). Pneumococci did not induce caspase 1-induced pyroptosis in less-differentiated human monocytes/macrophages under conditions where several Gram-negative bacteria were shown to do so (66). We also confirmed that alveolar macrophage PCD is unaltered in caspase 1^−/−^ murine alveolar macrophages following pneumococcal infection, confirming the PCD is not caspase 1 dependent. Progressive LMP, however, allows release of lysosomal proteases that prime apoptosis (58). We have previously shown cathepsin D activates MOMP, caspase 3 activation, and nuclear fragmentation in macrophages, all characteristics of apoptosis, during pneumococcal infection (20) and once again confirm here that the PCD resembles apoptosis. Cathepsin D also upregulates antioxidants, limiting lysosomal membrane peroxidation and the more extensive LMP associated with necrosis (20).

PLY contributes to LMP, but a functional requirement for LMP in apoptosis induction in macrophages phagocytosing *S. pneumoniae* has not previously been demonstrated. Like PLY, LMP is necessary but also not sufficient for induction of apoptosis. The extent of LMP determines the type of PCD (67, 68), and in our model, LMP is selective, allowing sensitization to apoptosis (58). The second role for PLY appears to be performed in the context of bacterial internalization and LMP, where it ensures engagement of apoptosis. We have shown previously that cathepsin D activation and induction of apoptosis represents a core component of the antimicrobial host defense of macrophages (20). This can be viewed as fitting broadly with PLY’s intracellular role as a stimulus for the induction of intracellular antimicrobial strategies, which we show also includes production of NO. Significantly, PLY contributes to the execution phase of apoptosis downstream of LMP and stimulates apoptosis in contrast to necrosis following induction of LMP with a lysomotrophic detergent. A number of studies illustrated how phagolysosomal cargo helps define the organelle’s interaction with other endosomes and ultimately, therefore, with the proteins it contains and how these are activated (69–71). Cathepsin D activation is critical in initiating the apoptotic cascade post-LMP (20, 72), and we now show that optimal activation of cathepsin D requires both phagocytosis of bacteria and PLY. PLY could influence the trafficking of lysosomes to the phagosome containing pneumococci or influence the resulting activation of cathepsin D to ensure induction of apoptosis and prevent alternative nonapoptotic PCD. Induction of apoptosis may also be viewed as a strategy that downregulates the inflammatory response, since LMP would otherwise allow ongoing inflammasome activation if the cell remained viable.

Our data therefore suggest a novel model in which PLY, acting in conjunction with other bacterial factors, first induces LMP by a mechanism independent of phagocytosis but involving PRR in activated cytokine-producing macrophages (see Fig. S9 in the supplemental material). This provides a novel host-mediated mechanism of LMP in response to a microorganism. The second stage of the host response to PLY ensures that the death process executed in response to LMP in the presence of intracellular bacteria is apoptosis, which requires cathepsin D activation (20). These results extend our understanding of the multifaceted roles of PLY as a focal point of the innate immune response to pneumococci that fine-tunes the host-pathogen interaction to ensure optimization of microbial killing and limitation of inflammation. Since this response is conserved among a range of PLY-related toxins and potentially involves an overlapping range of PRRs, it is likely to extend to many other microbial factors. Microbial clearance by tissue macrophages, such as the alveolar macrophage, is a critical bottleneck in innate host defense and represents a tipping point for the escalation of inflammatory responses (22). Models such as this can provide a rational basis for approaches that refocus the innate response at crucial tipping points and can enable us to develop host-focused approaches to treatment of common bacterial infections. These results suggest that the combination of the use of lysomotrophic agents, with the potential to selectively permeabilize the phagolysosome, and engagement of select signaling pathways downstream of this could have utility as part of this strategy.

## MATERIALS AND METHODS

### Bacteria.

Serotype 2 (D39 strain; NCTC 7466), mutant strains (D39Δ6 [Δ6] and D39-PLYSTOP [Stop]), and serotype 1 clinical isolates ST227 (strain INV104B isolated from a patient with invasive pneumococcal disease [37]) and ST306 (strain 03-2038 isolated from a patient with empyema from the Scottish Haemophilus, Legionella, Meningococcus and Pneumococcus Reference Laboratory [39]) of *S. pneumoniae* were grown as previously described, as was *S. mitis* (strain R751) (21). Mutant strains pAHS3 (expressing wild-type [WT] full-length [FL] pneumolysin), pAHS1 (expressing domains 1 to 3 [D1 to D3]), pAHS2 (expressing domain 4 [D4]), and pAHS6 (expressing WT RFP-tagged pneumolysin [RFPPLY]) (see Text S1 in the supplemental material for details of construction) were grown similarly but in the presence of 1 µg/ml erythromycin to maintain the plasmid. Prior to infection with *S. pneumoniae*, thawed aliquots were opsonized in RPMI medium (Sigma-Aldrich) containing 10% (vol/vol) anti-pneumococcal immune serum (21). Bacterial numbers were assessed by the surface viable count method after inoculation of bacteria on blood agar. All strains were verified by the optochin disc test and by Western blot analysis for PLY.

### Hemolytic assay.

Hemolytic activity of bacterial strains was assessed by analyzing hemoglobin release from defibrinated sheep blood (TCS Biosciences), as previously described (50).

### Cells and infection.

Human monocyte-derived macrophages (MDMs) were isolated from whole blood donated by healthy volunteers as previously described, with written informed consent as approved by the South Sheffield Regional Ethics Committee (21). Murine bone marrow-derived macrophages from C57BL/6, NALP3^−/−^, ASC^−/−^, TLR2^−/−^, TLR4^−/−^, or MyD88^−/−^ mice were isolated and cultured as previously described (20). Cells were incubated with opsonized pneumococci at a multiplicity of infection of 10 or mock infected as previously described (21). Infections involving strains pAHS1, pAHS2, pAHS3, and pAHS6 took place in the presence of 1 µg/ml erythromycin. All bacterial strains were internalized equally (see Fig. S1 in the supplemental material). In some experiments, macrophages were treated with 2 µg/ml cytochalasin D (Sigma) to inhibit bacterial internalization. In some experiments, cells were exposed to TLR2 blocking antibody (Ab) (clone TL2.1; eBioscience) (1:500) after preblocking for 30 min with 100 µg/ml IgG1 (Sigma) or the TLR4 antagonist lipopolysaccharide (LPS) from the photosynthetic bacterium *Rhodobacter sphaeroides* (Ultrapure LPS-RS) (InvivoGen) at 5 µg/ml. The efficacy of blocking TLR2 or TLR4 was tested by showing 96% and 97% reductions in TNF-αproduction in cells stimulated with 1 µg/ml lipoteichoic acid (LTA) and 10 ng/ml LPS, respectively. All reagents were added for 30 min prior to challenge with bacteria. In some experiments, macrophages were exposed to exogenous pneumolysin provided by T. Mitchell. Exogenous PLY contained <0.6 endotoxin units per μg protein.

### Bacterial internalization.

Assessment of intracellular killing was carried out at designated times using a gentamicin protection assay as previously described (23).

### Analysis of loss of lysosomal acidification.

To detect loss of lysosomal acidification, cells were stained with 5 µM of the azurophilic dye acridine orange (Sigma-Aldrich) (20) before being analyzed by flow cytometry.

### Lysosomal staining.

Macrophages were loaded with 1 µM pepstatin A-BODIPY FL conjugate (Invitrogen) in complete media, for 2 h at 37°C, before being washed in phosphate-buffered saline (PBS) and left overnight. Cells were imaged using a 40× objective on a fluorescence microscope (Leica DMI4000B) at an excitation value of 476 nm and emission values of 500 to 600 nm.

### Analysis of lysosomal leak.

Lysosomal rupture was assessed using FITC-dextran molecules (Sigma) (10-, 40-, and 250-kDa molecular mass). In brief, cells were incubated with 5 mg/ml FITC-dextran molecules for 2 h at 37°C. The cells were washed and chased with culture medium for 3 h (to allow colocalization with lysosomes [73]) before being challenged with bacteria in the normal manner. At the designated time postinfection, cells were visualized using a 100× objective and a confocal microscope (Zeiss LSM510 NLO Inverted), and the percentage of cells with diffuse staining was recorded (73).

### Permeabilization of lysosomes.

Permeabilization of macrophage lysosomes was achieved using the lysomotrophic detergent l-leucyl-l-leucine methyl ester (LeuLeuOMe) (Bachem). Macrophages were incubated with 1 mg/ml of LeuLeuoMe to induce permeabilization (66). In Stop-challenged macrophages, to ensure that permeabilization occurred at approximately the same time as in cells challenged with wild-type bacteria, LeuLeuOMe was added 6 h postchallenge.

### SDS-PAGE and Western immunoblotting.

Cytosolic and membrane fractions were isolated using digitonin and were subjected to Western blotting as previously described (20). Blots were blocked and incubated overnight at 4°C with antibodies against PLY (rabbit monoclonal; Abcam) (1:1,000), lysosome-associated membrane protein 1 (LAMP-1) (mouse monoclonal; BD Biosciences) (1:1,000), cathepsin B (rabbit polyclonal; Abcam) (1:1,000), cytochrome *c* (mouse monoclonal; BD Pharmingen) (1:1,000), RIP1 (rabbit polyclonal; Abcam) (1:1,000), RIP3 (rabbit polyclonal; Abcam) (1:1,000), or actin (rabbit polyclonal; Sigma) (1:5,000). Detection was performed with horseradish peroxidase (HRP)-conjugated goat anti-rabbit and anti-mouse immunoglobulins (Dako) and enhanced chemiluminescence (Amersham Pharmacia). Bands were quantified using ImageJ 1.32 software (NIH). Fold change from mock-infected or Stop-infected cell results was calculated and normalized to the corresponding fold change seen with actin.

### Caspase activity assays.

Cellular caspase activity was measured using a fluorescent caspase 3 or caspase 9 assay (Abcam) according to the manufacturer’s instructions. Fluorescence was measured on a Flash Varioskan (Thermo Scientific).

### Flow cytometry.

Flow cytometric measurements were performed using a four-color FACSCalibur flow cytometer (Becton, Dickinson). Forward- and side-scatter light was used to identify cell populations by size and granularity. In all experiments, 10,000 events were captured and analyzed with FlowJo software version 9.3.2 (Tree Star, Inc.).

### Detection of apoptosis.

To detect loss of Δψ_m_, at the required time points, cells were stained with 10 µM 5,5′,6,6′-tetrachloro-1,1′,3,3′ tetraethylbenzimidazolocarbocyanine iodide (JC-1; Sigma-Aldrich) and analyzed by flow cytometry. Loss of Δψ_m_ was demonstrated by a loss of fluorescence on the FL-2 channel as previously described (45). Nuclear fragmentation was detected by the use of 4′6′-diamidino-2-phenylindole (DAPI; Molecular Probes) staining as described previously (21).

### LDH assay.

The release of lactate dehydrogenase was measured using a Cytotox 96 cell viability kit (Promega) according to the instructions of the manufacturer.

### Statistics.

Results are presented as means and standard errors of the means, with the number of individual donors used in each data set shown as the “*n*” value. Differences between groups of treatments were calculated by analysis of variance (ANOVA) (Bonferroni’s posttest), using GraphPad Prism 5 (GraphPad Software, Inc.). Significance was defined as *P* = <0.05.

## SUPPLEMENTAL MATERIAL

Text S1 Supplemental methods. Download Text S1, DOCX file, 0.1 MB

Figure S1 Assessment of *S. pneumoniae* strains. Wild-type *S. pneumoniae* (D39), a D39 mutant expressing noncytolytic pneumolysin (Δ6), a pneumolysin-deficient D39 mutant (Stop), or reconstituted mutants expressing full-length pneumolysin (FL), pneumolysin domains 1 to 3 (D1-3), pneumolysin domain 4 only (D4), or red-fluorescent protein (RFP)-tagged pneumolysin were assessed for PLY protein expression and hemolytic ability. (A) Bacteria were lysed before being probed with anti-pneumolysin antibody. The antibody was not capable of detecting D4 only. (B) Red blood cells were incubated with bacterial lysate from the designated strain before optical density was measured. An increase in optical density equates to increased hemolytic activity. The negative (–ve) control was PBS, and the positive (+ve) control was water (*n* = 3). * = *P* < 0.05, ** = *P* < 0.01, *** = *P* < 0.001 (one-way ANOVA). Data are expressed as means ± SEM. (C) Monocyte-derived macrophages (MDM) were challenged with wild-type *S. pneumoniae* (D39), a D39 mutant expressing noncytolytic pneumolysin (Δ6), a pneumolysin-deficient D39 mutant (Stop), or reconstituted mutants expressing full-length pneumolysin (FL), pneumolysin domains 1 to 3 (D1-3), pneumolysin domain 4 only (D4), or red-fluorescent protein (RFP)-tagged pneumolysin. At 4 h postchallenge, numbers of viable internalized bacteria were assessed (*n* = 4.) No significant differences were found by one-way ANOVA. Data are expressed as means ± SEM. Download Figure S1, TIF file, 0.5 MB

Figure S2 Exogenous pneumolysin does not induce key steps in the apoptotic pathway, but internalized pneumolysin is required for maximal cathepsin D activation. Exogenous pneumolysin at the indicated concentration was incubated with monocyte-derived macrophages (MDM) for 16 h. (A and B) Cells were assessed for loss of lysosomal acidification (LLA) (A) or loss of inner mitochondrial transmembrane potential (Δψ_m_) (B) by flow cytometry. *n* = 4, no significant difference by one-way ANOVA. Data are expressed as means ± SEM. (C) MDMs were mock infected (MI) or challenged with wild-type *S. pneumoniae* (D39), 5 µg/ml pneumolysin (PLY), pneumolysin-deficient D39 (Stop), or Stop with exogenous pneumolysin (Stop + PLY) in the presence (+) or absence (-) of cytochalasin D. At 8 h postchallenge, cells were assessed for activation of cathepsin D (*n* = 3). * = *P* < 0.05 for MI versus D39 and for D39 versus PLY in cytochalasin D samples. Data are represented as means ± SEM. Download Figure S2, TIF file, 0.3 MB

Figure S3 Macrophages challenged with *Streptococcus pneumoniae* undergo a death process with characteristics of apoptosis. (A to C) Monocyte-derived macrophages (MDMs) were mock infected (MI) or challenged with wild-type *Streptococcus pneumoniae* (D39). At 20 h postchallenge, cells were lysed and the cytosolic fraction probed for cytochrome *c* by Western blotting (A), analyzed for cathepsin 9 activity (B), analyzed for cell membrane permeabilization using propidium iodide (PI) (C), or nuclear fragmentation using DAPI (D). For all experiments, *n* = 4. * = *P* < 0.05 (paired *t* test). Download Figure S3, TIF file, 0.3 MB

Figure S4 Nonhemolytic clinical isolates of *S. pneumoniae* and *S. mitis* induce levels of loss of lysososomal acidification, loss of inner mitochondrial transmembrane potential, and apoptosis comparable to those seen with wild-type *S. pneumoniae*. (A) Monocyte-derived macrophages (MDMs) were mock infected (MI) or challenged with the NOD2 agonist muramyldipeptide (MDP) or the NOD1/2 agonist MTri_DAP_ (MurNAc-l-Ala-d-Glu-meso-diaminopimelic acid) in the presence (+) or absence (-) of 5 µg/ml exogenous pneumolysin (PLY). At 16 h postchallenge, cells were analyzed for loss of lysosomal acidification (LLA) by flow cytometry. (B and C) MDMs were mock infected (MI) or challenged with wild-type *S. pneumoniae*, serotype 2 *S. pneumoniae* (D39), a hemolytic serotype 1 *S. pneumoniae* strain (ST227), or a nonhemolytic serotype 1 *S. pneumoniae* strain (ST306). At 16 h postchallenge, cells were analyzed for LLA (B) or loss of inner mitochondrial transmembrane potential (Δψ_m_) (C) (*n* = 3). ns, not significant (one-way ANOVA). Data are represented as means ± SEM. (D to F) MDMs were MI or challenged with D39, *Streptococcus mitis* (*S. mitis*), or pneumolysin-deficient *S. pneumoniae* (Stop). At 16 h postchallenge, the cells were assessed for LLA (D) or for loss of Δψ_m_ (E). (F) At 20 h postchallenge, cells were assessed for nuclear fragmentation. For panels D to F, *n* = 4. ns = not significant, * = *P* < 0.05, ** = *P* < 0.01, *** = *P* < 0.001 (one-way ANOVA). Data are expressed as means ± SEM. Download Figure S4, TIF file, 0.4 MB

Figure S5 NLRP3 and ASC are not involved in the induction of loss of lysosomal acidification or apoptosis in response to pneumolysin. Bone marrow-derived macrophages (BMDM) from wild-type C57BL/6 (BL6), cytosolic Nod-like receptor family, pyrin domain-containing protein 3-deficient (NLRP3^−/−^), or apoptosis-associated speck-like protein containing a caspase recruitment domain-deficient (ASC^−/−^) mice were mock infected (MI) or challenged with 5 µg/ml exogenous pneumolysin (5PLY), wild-type *S. pneumoniae* (D39), or pneumolysin-deficient *S. pneumoniae* (Stop). (A to C) Cells were assessed for loss of lysosomal acidification (LLA) (A) and loss of inner mitochondrial transmembrane potential (Δψ_m_) (B) at 16 h postchallenge and assessed for nuclear fragmentation at 20 h postchallenge (C). In all experiments, *n* = 3 to 4 per group. No significant differences between wild-type and knockout mice were seen under any conditions by two-way ANOVA. Data are expressed as means ± SEM. (D) C57BL/6 wild-type (WT) or caspase 1^−/−^ mice were MI or challenged with serotype 1 *S. pneumoniae* (Spn). At 24 h postchallenge, alveolar macrophages (AM) were obtained and assessed for apoptosis by nuclear fragmentation (*n* = 6 to 11 mice per group). Download Figure S5, TIF file, 0.4 MB

Figure S6 Effect of pneumolysin and bacterial internalization on macrophage innate effector function. (A to F) Monocyte-derived macrophages (MDM) were mock infected (MI) or challenged with either wild-type *S. pneumoniae* (D39) or pneumolysin-deficient D39 (Stop) in the presence (+) or absence (-) of cytochalasin D (CD) or 5 µg exogenous pneumolysin (PLY). (A to D) At the designated time postchallenge, cells were analyzed for the production of the cytokines IL-1β, TNF-α, IL-8, and IL-6. (E) MDMs were MI or challenged with the designated strain of D39 in the presence (+) or absence of PLY. At 8 h postchallenge, cells were incubated with fluorescent latex beads for a further 2 h before bead internalization was measured by flow cytometry measuring median fluorescence intensity (MFI). (F and G) MDMs were studied under the same conditions as those used for the experiments described for panels A to D, and levels of reactive oxygen species (ROS) (F) or nitric oxide (NO) (G) were measured. In all experiments, *n* = 3 to 4. * = *P* < 0.05, *** = *P* < 0.001 (one-way ANOVA within each time point). Data are expressed as means ± SEM. Download Figure S6, TIF file, 0.5 MB

Figure S7 Exposure of macrophages to pneumolysin-deficient bacteria and LeuLeuOMe results in levels of LLA and LMP similar to those seen after challenge with wild-type *S. pneumoniae*. (A) Monocyte-derived macrophages (MDM) were challenged with the specified dose (μg/ml) of the lysomotropic detergent LeuLeuOMe. At the designated time postchallenge, cells were analyzed for loss of lysosomal acidification (LLA) by flow cytometry (*n* = 3). (B) MDMs were mock infected (MI) or challenged with either wild-type *S. pneumoniae* (D39) or pneumolysin-deficient D39 (Stop). Some cells were challenged with Stop in the presence of the lysomotropic detergent LeuLeuOMe (Stop + Leu). At 16 h postchallenge, cells were analyzed for LLA (*n* = 5). ns = not significant, * = *P* < 0.05 (one-way ANOVA). (C) Macrophages were either MI or challenged with D39 or with Stop. Some macrophages were challenged in the presence of 5 µg/ml pneumolysin (PLY) or LeuLeuOMe. At 16 h postchallenge, cytosolic fractions were obtained and probed for the lysosomal protein cathepsin B. Western blots are representative of the results of three independent experiments. Densitometry was carried out, and fold change in Cat B relative to Stop-challenged cells was calculated (*n* = 3). Data are expressed as means ± SEM. Download Figure S7, TIF file, 0.5 MB

Figure S8 The presence of pneumolysin modifies the type of macrophage death. (A) Monocyte-derived macrophages (MDM) were mock infected (MI; white bar) or challenged with either wild-type *S. pneumoniae* (D39; black bar) or pneumolysin-deficient D39 (Stop; gray bar). Cells were challenged in the presence (+) or absence (-) of the lysomotrophic detergent LeuLeuOMe, cytochalasin D (CD), or 5 µg/ml exogenous pneumolysin (PLY) (*n* = 5). * = *P* < 0.05, ** = *P* < 0.01, *** = *P* < 0.001 (one-way ANOVA). (B) MDMs were challenged as described for panel A but in either the absence (-) or the presence (+) of 30 nM necrostatin. At 20 h postchallenge, cells were assessed for LDH release (*n* = 3). (C) MDMs were mock infected (MI) or challenged with the designated strain of bacteria in the presence (+) or absence (-) of LeuLeuOMe and/or 5 µg/ml pneumolysin (+PLY). At 20 h postchallenge, cells were analyzed and probed for receptor-interacting serine/threonine protein kinase 1 (RIP1) and RIP3. For the positive control (+ve), cells were incubated with 5 nM TNF-α and 10 µM zVAD-fmk for 16 h. Blots are representative of the results of three independent experiments. (D) MDMs were either mock infected (MI) or challenged with D39, a D39 mutant expressing noncytolytic pneumolysin (Δ6), Stop, or reconstituted mutants expressing full-length pneumolysin (FL), pneumolysin domains 1 to 3 (D1-3), pneumolysin domain 4 only (D4), or red fluorescent protein-tagged pneumolysin (RFP). At 20 h postchallenge, cells were assessed for necrosis by measuring LDH release (*n* = 6). *** = *P* < 0.001 (one-way ANOVA). All data are expressed as means ± SEM. Download Figure S8, TIF file, 0.8 MB

Figure S9 Model of lysosomal membrane permeabilization in *S. pneumoniae*-challenged macrophages. Pneumolysin (PLY) acts in two stages to stimulate apoptosis. In the first stage (1), the early phase of macrophage sensitization for apoptosis involves PLY, which, acting independently of its pore-forming capacity but in concert with other microbial factors, induces lysosomal/phagolysosomal membrane permeabilization (LMP), in a process that is independent of bacterial phagocytosis. This process involves Toll-like receptor 2 (TLR2) and TLR4 and potentially other signals generated through additional pattern recognition receptors (PRRs). Intracellular bacteria release PLY during LMP, which progressively accumulates in the cytosol, where it has the potential to engage additional unidentified pathways. Intracellular PLY is required for maximal activation of cathepsin D and for engagement of the second phase of apoptosis (2) through execution of the apoptosis program downstream of LMP. This involves mitochondrial outer membrane permeabilization, activation of caspase 9 and 3, and, ultimately, nuclear fragmentation, which ensures the death of the macrophage by apoptosis rather than necrosis. Phagocytosis-independent PLY signals induce LMP but are also associated with cytokine generation, while the phagocytosis-dependent responses induced by PLY that are associated with apoptosis induction are also associated with other aspects of antimicrobial host defense such as nitric oxide generation. Download Figure S9, TIF file, 0.9 MB
