# Supplementary material for: Pneumolysin Activates Macrophage Lysosomal Membrane Permeabilization and Executes Apoptosis by Distinct Mechanisms without Membrane Pore Formation
Source: mBio. 2014 Oct 7;5(5):e01710-14. doi: 10.1128/mBio.01710-14 (PMC4196231; doi:10.1128/mBio.01710-14)
Supplement: Text S1 — Supplemental methods. Download [file mbo005142021s1.docx]

**Supplemental Methods**

*Construction* of *D39Δ6 (Δ6), D39PLY-STOP (Stop), pAHS1 (D1-3), pAHS2 (D4), pAHS3 (FL) and pAHS6 (RFPPLY)*: The strain D39Δ6 was generated as described previously (Daigneault M, De Silva TI, Bewley MA, Preston JA, Marriott HM, et al. PLoS pathogens **8**: e1002814). PLY knockout strain D39PLY-STOP was created using the same strategy from the *S. pneumoniae* strain D39. The PLY gene was replaced with a PLY sequence in which a single T nucleotide was introduced after the 6th base in the original PLY sequence generating a stop codon after the second amino acid (Johnston CHG (2008). Glasgow.: University of Glasgow). The codon optimised sequence of PLY FL allele 2 from Jefferies *et al*. (Jefferies J, Nieminen L, Kirkham LA, Johnston C, Smith A, et al. (2007). J Bacteriol **189**: 627-632) was synthesized with the 3 tags His, FLAG and Lumio (FlAsH) at the N-terminal end by DNA 2.0 (California, USA). The constitutive promoter from the aminopterin resistance operon (ami) was added upstream (Claverys JP, Dintilhac A, Pestova EV, Martin B, Morrison DA (1995) Gene 164: 123-128) and the resulting fragment was then subcloned into the *E. coli*-Streptococcal shuttle vector pAL2YI (Ibrahim YM, Kerr AR, McCluskey J, Mitchell TJ (2004). Journal of bacteriology 186: 5258-5266) . Sub-cloning was used to create a number of further constructs replacing the FL PLY gene sequence with either domains 1-3 or domain 4. An additional N-terminus RFP tagged version of FL PLY was created. The resulting plasmids were pAHS1 containing PLY domains 123 with 3 tags; pAHS2 containing PLY domain 4 with 3 tags; pAHS3 containing complete PLY with 3 tags; pAHS6 containing RFP-Ply fusion with 3 tags. These plasmids were then transformed into *S. pneumoniae* strain D39PLY-STOP. All strains were assessed for PLY expression and hemolytic activity (Figure S1).

*Generation of bone-marrow-derived macrophages:* All animal experiments were performed in accordance with the UK Animals (Scientific procedures) Act, authorized under a UK Home Office License (40/3251), and approved by the animal project review committee of the University of Sheffield. Bone marrow-derived macrophages (BMDM) were isolated as previously described (Bewley MA, Marriott HM, Tulone C, Francis SE, Mitchell TJ, Read RC, Chain B, Kroemer G,et al. PLoS pathogens **7**:e1001262), using C57Bl6 mice (Harlan, Oxford UK) or mice deficient in NLRP3^-/-^ or ASC^-/-^ obtained from Millenium Pharmaceuticals (McNeela EA, Burke A, Neill DR, Baxter C, Fernandes VE, et al**.** PLoS pathogens **6**: e1001191) (Fang R, Tsuchiya K, Kawamura I, Shen Y, Hara H, et al. (2011) J Immunol **187**: 4890-4899) **(**Kanneganti TD, Ozoren N, Body-Malapel M, Amer A, Park JH, et al. Nature **440**: 233-236). BMDM were obtained by culturing bone marrow cells plated at 0.5 x 10^6^ cells/ml for 14 d in DMEM containing 10% FCS and 10% conditioned L929 media (Dockrell DH, Marriott HM, Prince LR, Ridger VC, Ince PG, et al. J Immunol **171**: 5380-5388). BMDM from all strains internalized bacteria at the same rate.

*Cathepsin D activity assay:* Cathepsin D activity was assessed using a fluorometric assay (Abcam) as per the manufacturers instructions. Fluorescence was measured on a Flash Varioskan (Thermo Scientific).

*Measuring apoptosis in caspase 1^-/-^ murine alveolar macrophages:* Alveolar macrophages were isolated from the lungs of C57BL/6 or caspase 1 ^-/-^ mice 24 h after infection with serotype 1 *S. pneumoniae* as previously described, with apoptosis detected on cytospins as described previously (Dockrell DH, Marriott HM, Prince LR, Ridger VC, Ince PG, et al. J Immunol **171**: 5380-5388).

*Caspase 1 inhibition:* In some experiments, macrophages were treated with 10μM of the caspase 1 inhibitor z-YVADfmk (Calbiochem). Macrophages were teated for 30 minutes prior to challenge with bacteria.

*Inhibition of necroptosis:* Necroptosis was inhibited by treating macrophages with 30nM of necrostatin (Sigma), added 30 minutes prior to challenge with bacteria.

*Cytokine generation*: Cells were challenged with the bacteria as described, and supernatants were harvested at the designated times, and stored at −80°C until analysis. IL-1β, TNF-α, IL-6 and IL-8 levels were measured by ELISA (R & D Systems) according to manufacturer's instructions.

*Measurement of reactive oxygen species and nitric oxide:* To measure NO, cells were incubated in phenol-red free RPMI containing 5 µM difluorescein diacetate (DAF-FM) (Sigma) as previously described (Marriott HM, Ali F, Read RC, Mitchell TJ, Whyte MK, Dockrell DH. 2004. FASEB **18:**1126-1128.) To measure reactive oxygen species (ROS) macrophages were incubated with 10 μM of the cell permeable molecule 2′, 7′-dichloro-dihydrofluorescein diacetate (DCF; Sigma-Aldrich) as previously described Hiraoka W, Vazquez N, Nieves-Neira W, Chanock SJ, Pommier Y. 1998. J Clin Invest **102**:1961-1968.

*NOD agonist treatment*: In some experiments cells were exposed to NOD1/NOD2 (MurNAc-L-Ala--D-Glu-meso-diaminopimelic acid, MTri_DAP_) and NOD2 (MurNAc-L-Ala-D-isoGln, MDP) agonists (10μg/ml, both InvivoGen).
