# Supplementary figures and images for: Pneumolysin Activates Macrophage Lysosomal Membrane Permeabilization and Executes Apoptosis by Distinct Mechanisms without Membrane Pore Formation
Source: mBio. 2014 Oct 7;5(5):e01710-14. doi: 10.1128/mBio.01710-14 (PMC4196231; doi:10.1128/mBio.01710-14)

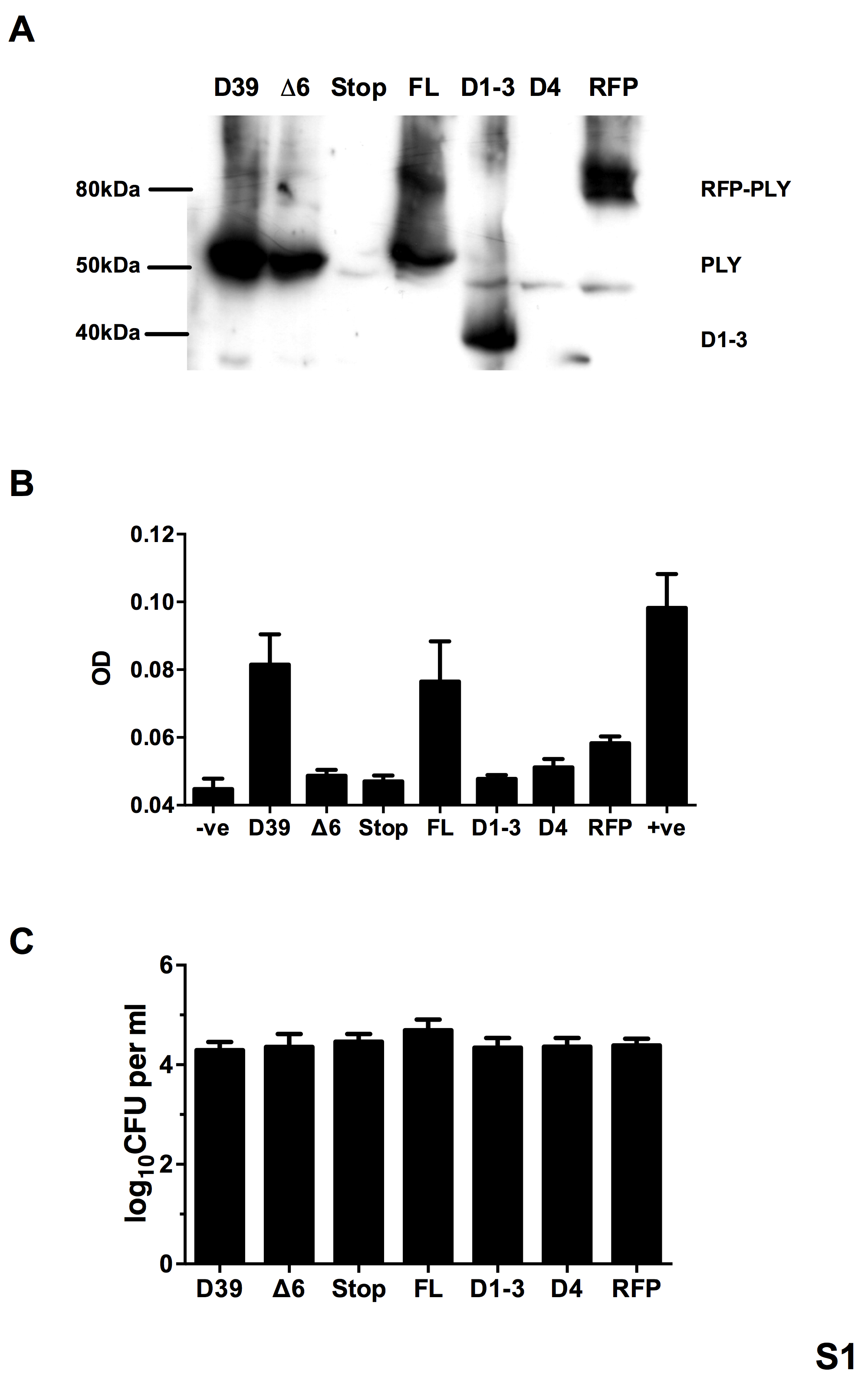

Supplement: Figure S1 — Assessment of S. pneumoniae strains. Wild-type S. pneumoniae (D39), a D39 mutant expressing noncytolytic pneumolysin (Δ6), a pneumolysin-deficient D39 mutant (Stop), or reconstituted mutants expressing full-length pneumolysin (FL), pneumolysin domains 1 to 3 (D1-3), pneumolysin domain 4 only (D4), or red-fluorescent protein (RFP)-tagged pneumolysin were assessed for PLY protein expression and hemolytic ability. (A) Bacteria were lysed before being probed with anti-pneumolysin antibody. The antibody was not capable of detecting D4 only. (B) Red blood cells were incubated with bacterial lysate from the designated strain before optical density was measured. An increase in optical density equates to increased hemolytic activity. The negative (–ve) control was PBS, and the positive (+ve) control was water (n = 3). * = P < 0.05, ** = P < 0.01, *** = P < 0.001 (one-way ANOVA). Data are expressed as means ± SEM. (C) Monocyte-derived macrophages (MDM) were challenged with wild-type S. pneumoniae (D39), a D39 mutant expressing noncytolytic pneumolysin (Δ6), a pneumolysin-deficient D39 mutant (Stop), or reconstituted mutants expressing full-length pneumolysin (FL), pneumolysin domains 1 to 3 (D1-3), pneumolysin domain 4 only (D4), or red-fluorescent protein (RFP)-tagged pneumolysin. At 4 h postchallenge, numbers of viable internalized bacteria were assessed (n = 4.) No significant differences were found by one-way ANOVA. Data are expressed as means ± SEM. Download [file mbo005142021sf1.tif]

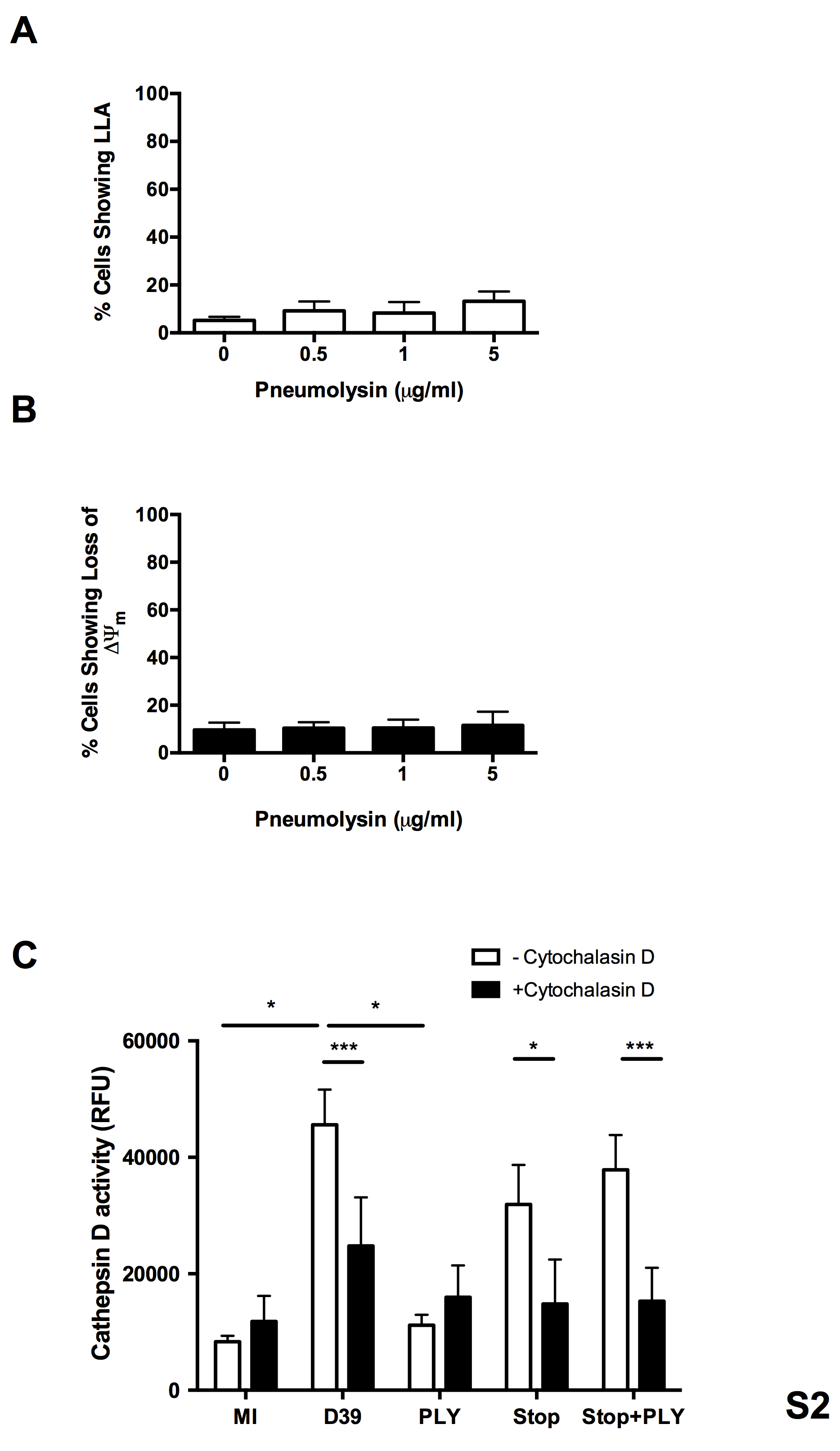

Supplement: Figure S2 — Exogenous pneumolysin does not induce key steps in the apoptotic pathway, but internalized pneumolysin is required for maximal cathepsin D activation. Exogenous pneumolysin at the indicated concentration was incubated with monocyte-derived macrophages (MDM) for 16 h. (A and B) Cells were assessed for loss of lysosomal acidification (LLA) (A) or loss of inner mitochondrial transmembrane potential (Δψm) (B) by flow cytometry. n = 4, no significant difference by one-way ANOVA. Data are expressed as means ± SEM. (C) MDMs were mock infected (MI) or challenged with wild-type S. pneumoniae (D39), 5 µg/ml pneumolysin (PLY), pneumolysin-deficient D39 (Stop), or Stop with exogenous pneumolysin (Stop + PLY) in the presence (+) or absence (-) of cytochalasin D. At 8 h postchallenge, cells were assessed for activation of cathepsin D (n = 3). * = P < 0.05 for MI versus D39 and for D39 versus PLY in cytochalasin D samples. Data are represented as means ± SEM. Download [file mbo005142021sf2.tif]

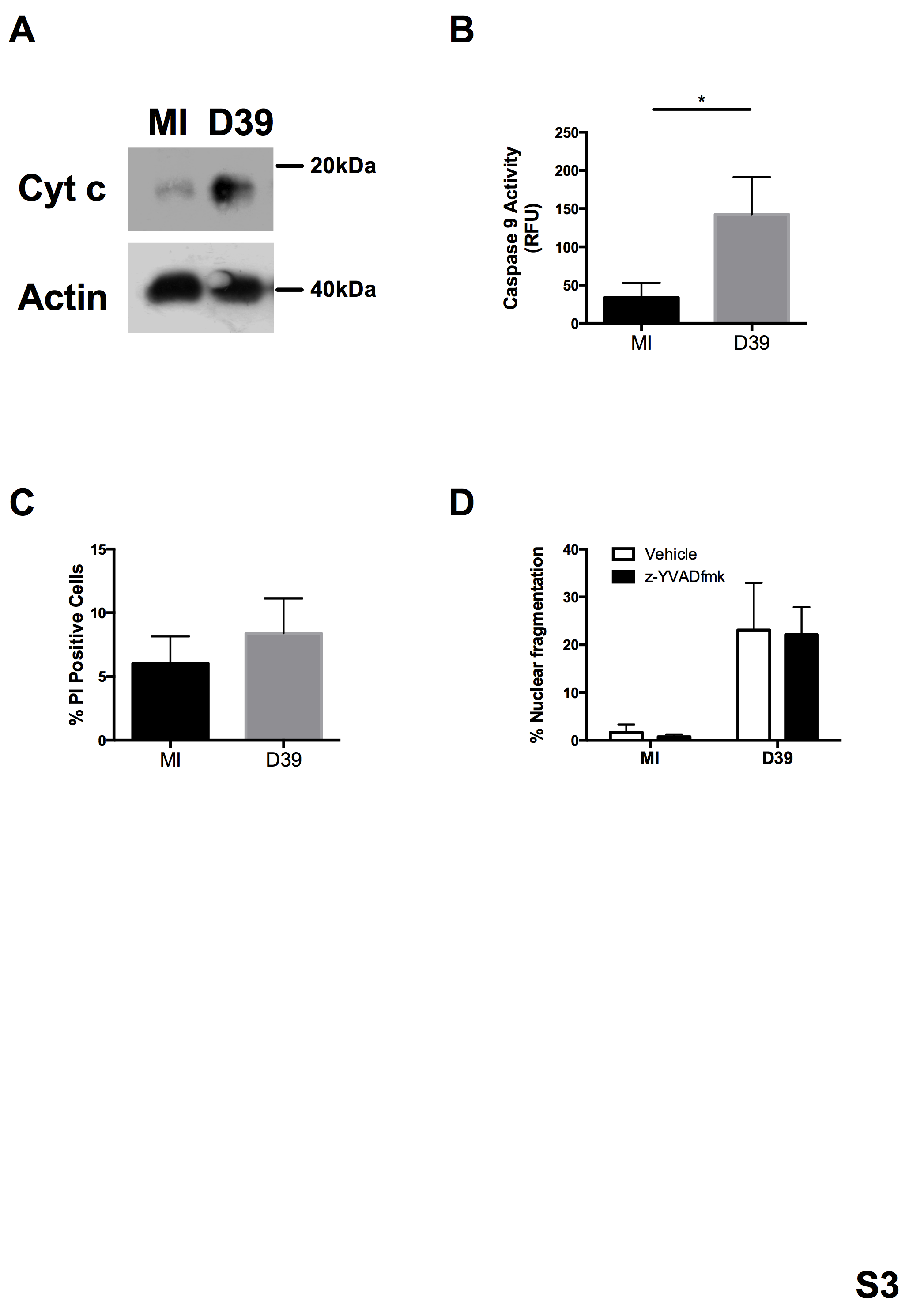

Supplement: Figure S3 — Macrophages challenged with Streptococcus pneumoniae undergo a death process with characteristics of apoptosis. (A to C) Monocyte-derived macrophages (MDMs) were mock infected (MI) or challenged with wild-type Streptococcus pneumoniae (D39). At 20 h postchallenge, cells were lysed and the cytosolic fraction probed for cytochrome c by Western blotting (A), analyzed for cathepsin 9 activity (B), analyzed for cell membrane permeabilization using propidium iodide (PI) (C), or nuclear fragmentation using DAPI (D). For all experiments, n = 4. * = P < 0.05 (paired t test). Download [file mbo005142021sf3.tif]

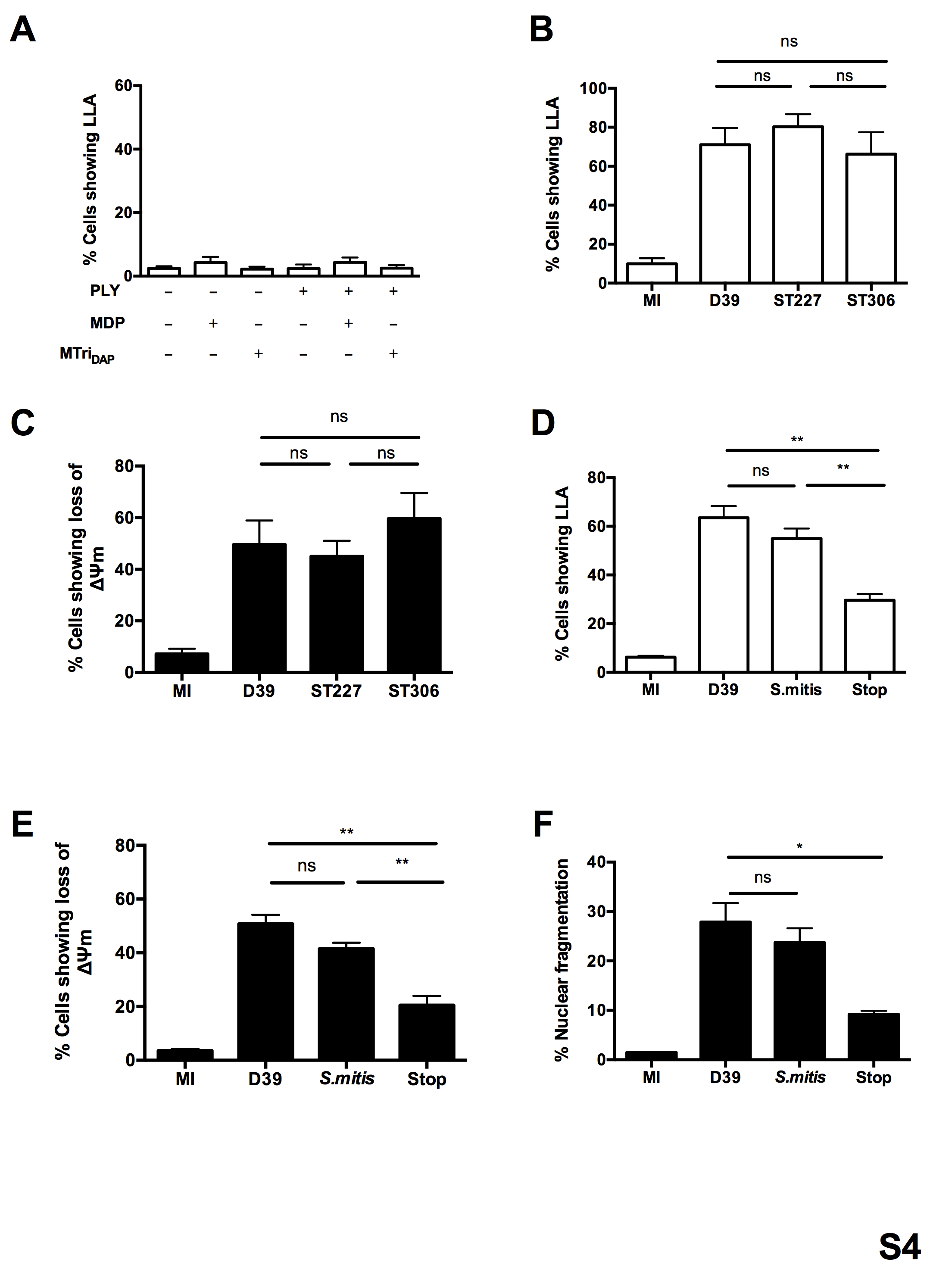

Supplement: Figure S4 — Nonhemolytic clinical isolates of S. pneumoniae and S. mitis induce levels of loss of lysososomal acidification, loss of inner mitochondrial transmembrane potential, and apoptosis comparable to those seen with wild-type S. pneumoniae. (A) Monocyte-derived macrophages (MDMs) were mock infected (MI) or challenged with the NOD2 agonist muramyldipeptide (MDP) or the NOD1/2 agonist MTriDAP (MurNAc-l-Ala-d-Glu-meso-diaminopimelic acid) in the presence (+) or absence (-) of 5 µg/ml exogenous pneumolysin (PLY). At 16 h postchallenge, cells were analyzed for loss of lysosomal acidification (LLA) by flow cytometry. (B and C) MDMs were mock infected (MI) or challenged with wild-type S. pneumoniae, serotype 2 S. pneumoniae (D39), a hemolytic serotype 1 S. pneumoniae strain (ST227), or a nonhemolytic serotype 1 S. pneumoniae strain (ST306). At 16 h postchallenge, cells were analyzed for LLA (B) or loss of inner mitochondrial transmembrane potential (Δψm) (C) (n = 3). ns, not significant (one-way ANOVA). Data are represented as means ± SEM. (D to F) MDMs were MI or challenged with D39, Streptococcus mitis (S. mitis), or pneumolysin-deficient S. pneumoniae (Stop). At 16 h postchallenge, the cells were assessed for LLA (D) or for loss of Δψm (E). (F) At 20 h postchallenge, cells were assessed for nuclear fragmentation. For panels D to F, n = 4. ns = not significant, * = P < 0.05, ** = P < 0.01, *** = P < 0.001 (one-way ANOVA). Data are expressed as means ± SEM. Download [file mbo005142021sf4.tif]

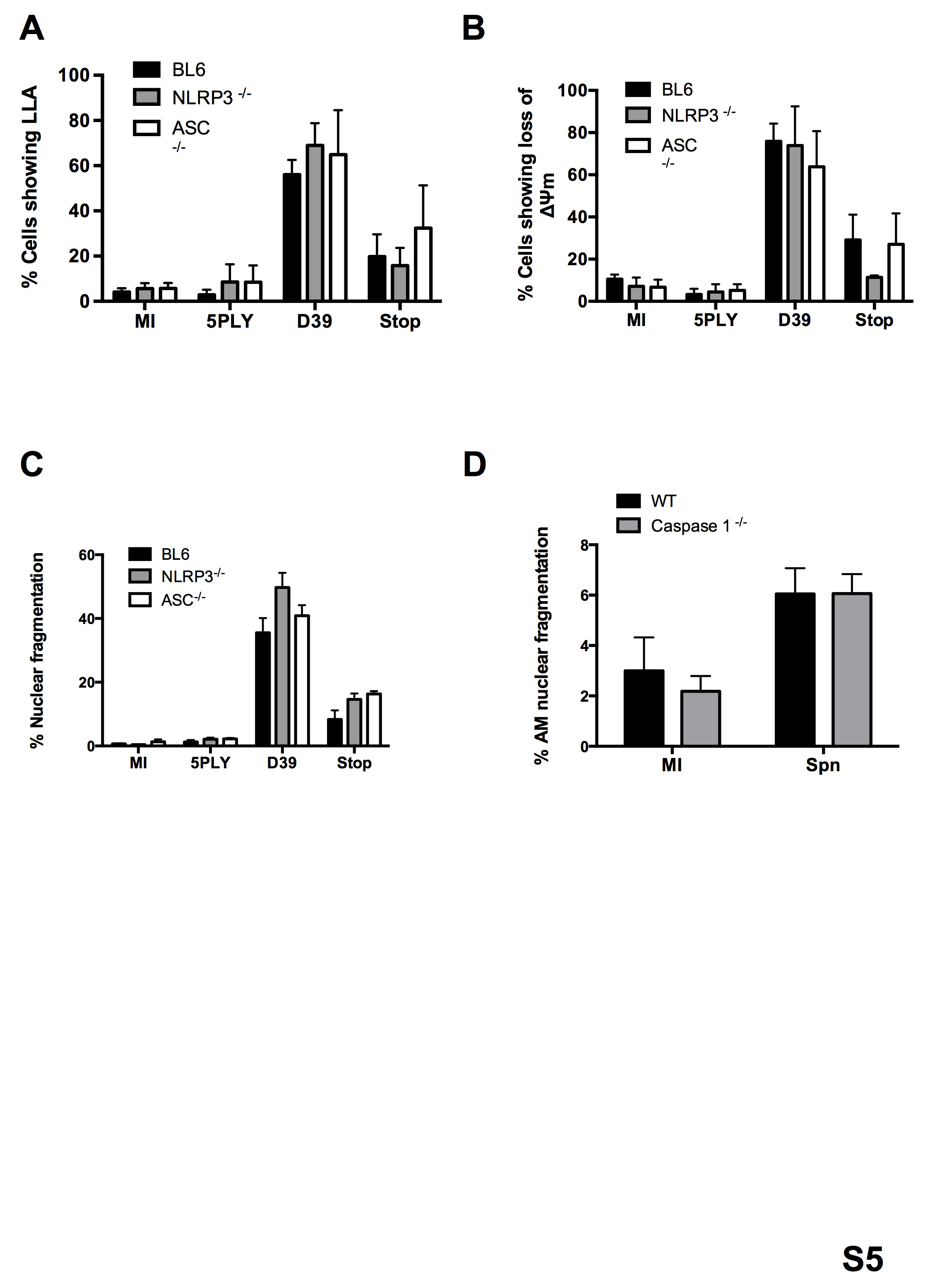

Supplement: Figure S5 — NLRP3 and ASC are not involved in the induction of loss of lysosomal acidification or apoptosis in response to pneumolysin. Bone marrow-derived macrophages (BMDM) from wild-type C57BL/6 (BL6), cytosolic Nod-like receptor family, pyrin domain-containing protein 3-deficient (NLRP3−/−), or apoptosis-associated speck-like protein containing a caspase recruitment domain-deficient (ASC−/−) mice were mock infected (MI) or challenged with 5 µg/ml exogenous pneumolysin (5PLY), wild-type S. pneumoniae (D39), or pneumolysin-deficient S. pneumoniae (Stop). (A to C) Cells were assessed for loss of lysosomal acidification (LLA) (A) and loss of inner mitochondrial transmembrane potential (Δψm) (B) at 16 h postchallenge and assessed for nuclear fragmentation at 20 h postchallenge (C). In all experiments, n = 3 to 4 per group. No significant differences between wild-type and knockout mice were seen under any conditions by two-way ANOVA. Data are expressed as means ± SEM. (D) C57BL/6 wild-type (WT) or caspase 1−/− mice were MI or challenged with serotype 1 S. pneumoniae (Spn). At 24 h postchallenge, alveolar macrophages (AM) were obtained and assessed for apoptosis by nuclear fragmentation (n = 6 to 11 mice per group). Download [file mbo005142021sf5.tif]

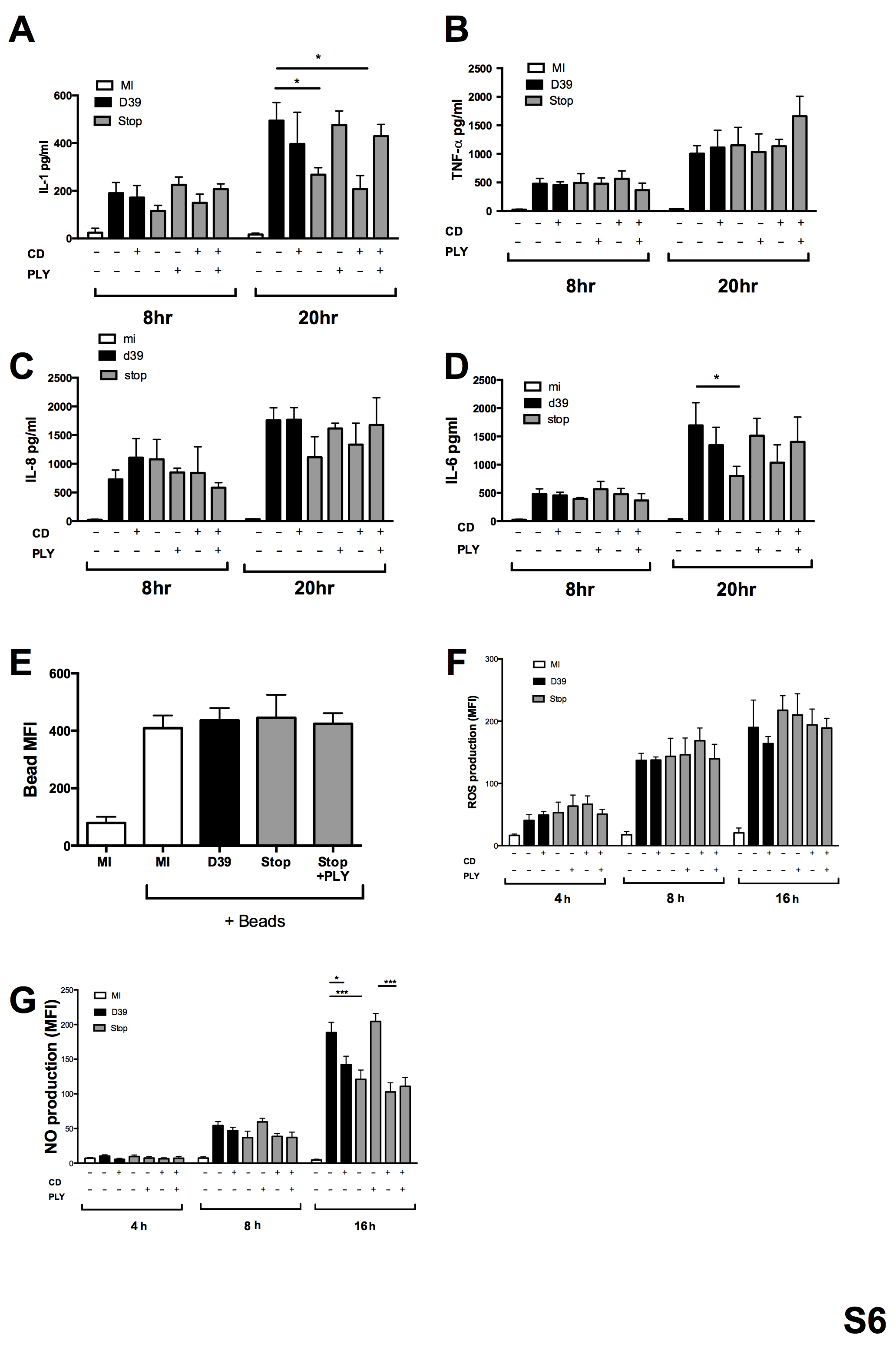

Supplement: Figure S6 — Effect of pneumolysin and bacterial internalization on macrophage innate effector function. (A to F) Monocyte-derived macrophages (MDM) were mock infected (MI) or challenged with either wild-type S. pneumoniae (D39) or pneumolysin-deficient D39 (Stop) in the presence (+) or absence (-) of cytochalasin D (CD) or 5 µg exogenous pneumolysin (PLY). (A to D) At the designated time postchallenge, cells were analyzed for the production of the cytokines IL-1β, TNF-α, IL-8, and IL-6. (E) MDMs were MI or challenged with the designated strain of D39 in the presence (+) or absence of PLY. At 8 h postchallenge, cells were incubated with fluorescent latex beads for a further 2 h before bead internalization was measured by flow cytometry measuring median fluorescence intensity (MFI). (F and G) MDMs were studied under the same conditions as those used for the experiments described for panels A to D, and levels of reactive oxygen species (ROS) (F) or nitric oxide (NO) (G) were measured. In all experiments, n = 3 to 4. * = P < 0.05, *** = P < 0.001 (one-way ANOVA within each time point). Data are expressed as means ± SEM. Download [file mbo005142021sf6.tif]

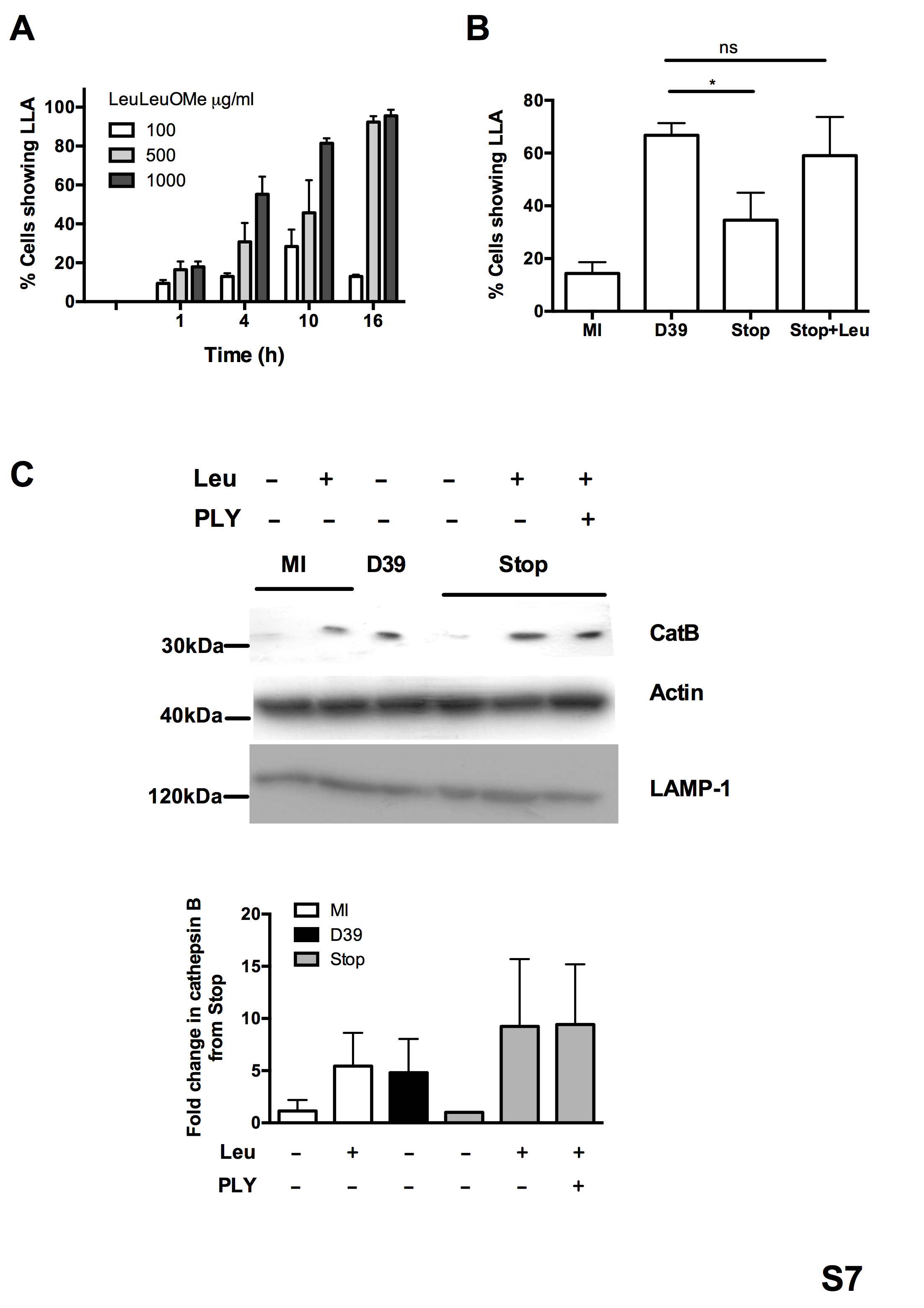

Supplement: Figure S7 — Exposure of macrophages to pneumolysin-deficient bacteria and LeuLeuOMe results in levels of LLA and LMP similar to those seen after challenge with wild-type S. pneumoniae. (A) Monocyte-derived macrophages (MDM) were challenged with the specified dose (μg/ml) of the lysomotropic detergent LeuLeuOMe. At the designated time postchallenge, cells were analyzed for loss of lysosomal acidification (LLA) by flow cytometry (n = 3). (B) MDMs were mock infected (MI) or challenged with either wild-type S. pneumoniae (D39) or pneumolysin-deficient D39 (Stop). Some cells were challenged with Stop in the presence of the lysomotropic detergent LeuLeuOMe (Stop + Leu). At 16 h postchallenge, cells were analyzed for LLA (n = 5). ns = not significant, * = P < 0.05 (one-way ANOVA). (C) Macrophages were either MI or challenged with D39 or with Stop. Some macrophages were challenged in the presence of 5 µg/ml pneumolysin (PLY) or LeuLeuOMe. At 16 h postchallenge, cytosolic fractions were obtained and probed for the lysosomal protein cathepsin B. Western blots are representative of the results of three independent experiments. Densitometry was carried out, and fold change in Cat B relative to Stop-challenged cells was calculated (n = 3). Data are expressed as means ± SEM. Download [file mbo005142021sf7.tif]

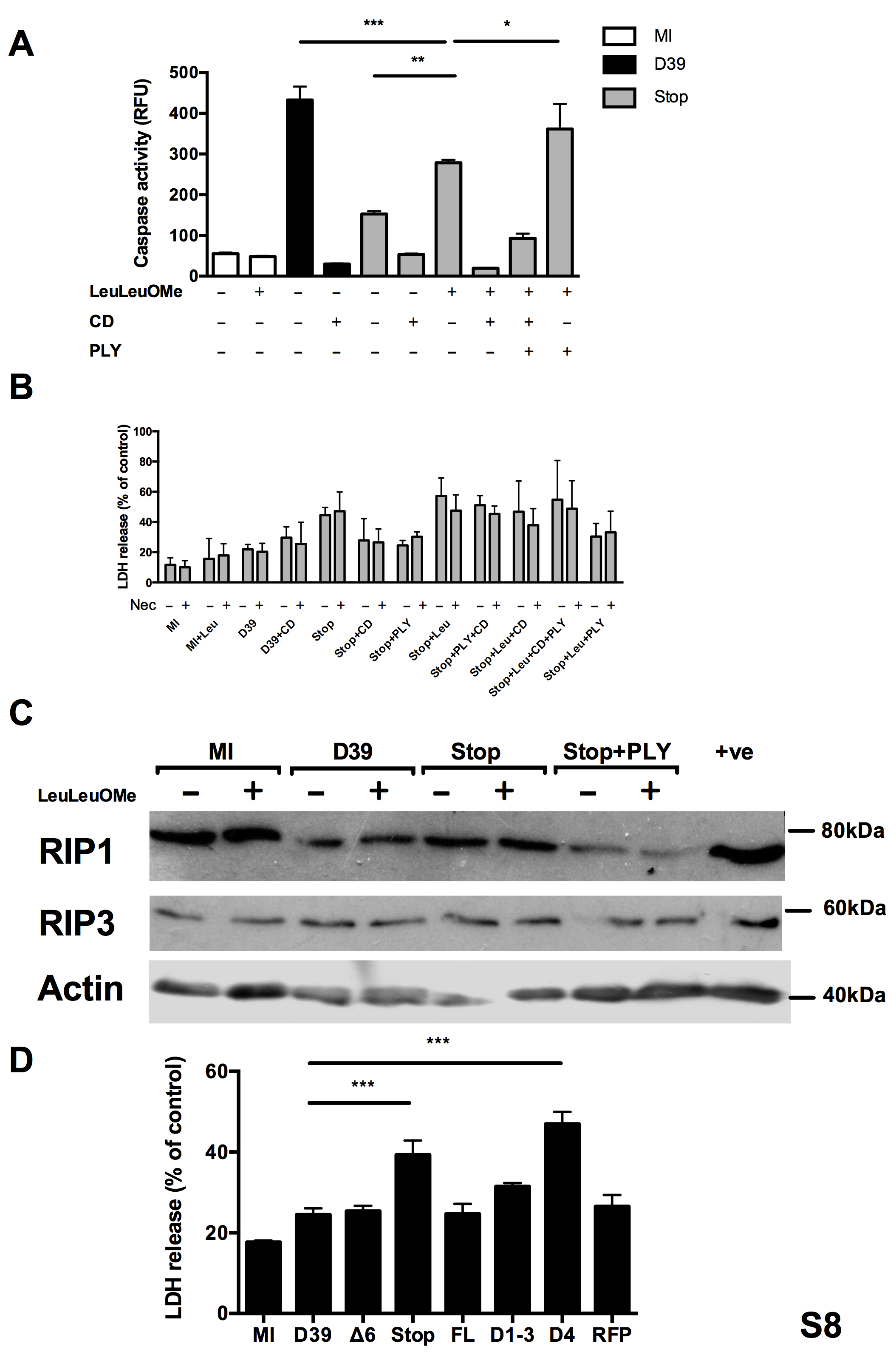

Supplement: Figure S8 — The presence of pneumolysin modifies the type of macrophage death. (A) Monocyte-derived macrophages (MDM) were mock infected (MI; white bar) or challenged with either wild-type S. pneumoniae (D39; black bar) or pneumolysin-deficient D39 (Stop; gray bar). Cells were challenged in the presence (+) or absence (-) of the lysomotrophic detergent LeuLeuOMe, cytochalasin D (CD), or 5 µg/ml exogenous pneumolysin (PLY) (n = 5). * = P < 0.05, ** = P < 0.01, *** = P < 0.001 (one-way ANOVA). (B) MDMs were challenged as described for panel A but in either the absence (-) or the presence (+) of 30 nM necrostatin. At 20 h postchallenge, cells were assessed for LDH release (n = 3). (C) MDMs were mock infected (MI) or challenged with the designated strain of bacteria in the presence (+) or absence (-) of LeuLeuOMe and/or 5 µg/ml pneumolysin (+PLY). At 20 h postchallenge, cells were analyzed and probed for receptor-interacting serine/threonine protein kinase 1 (RIP1) and RIP3. For the positive control (+ve), cells were incubated with 5 nM TNF-α and 10 µM zVAD-fmk for 16 h. Blots are representative of the results of three independent experiments. (D) MDMs were either mock infected (MI) or challenged with D39, a D39 mutant expressing noncytolytic pneumolysin (Δ6), Stop, or reconstituted mutants expressing full-length pneumolysin (FL), pneumolysin domains 1 to 3 (D1-3), pneumolysin domain 4 only (D4), or red fluorescent protein-tagged pneumolysin (RFP). At 20 h postchallenge, cells were assessed for necrosis by measuring LDH release (n = 6). *** = P < 0.001 (one-way ANOVA). All data are expressed as means ± SEM. Download [file mbo005142021sf8.tif]

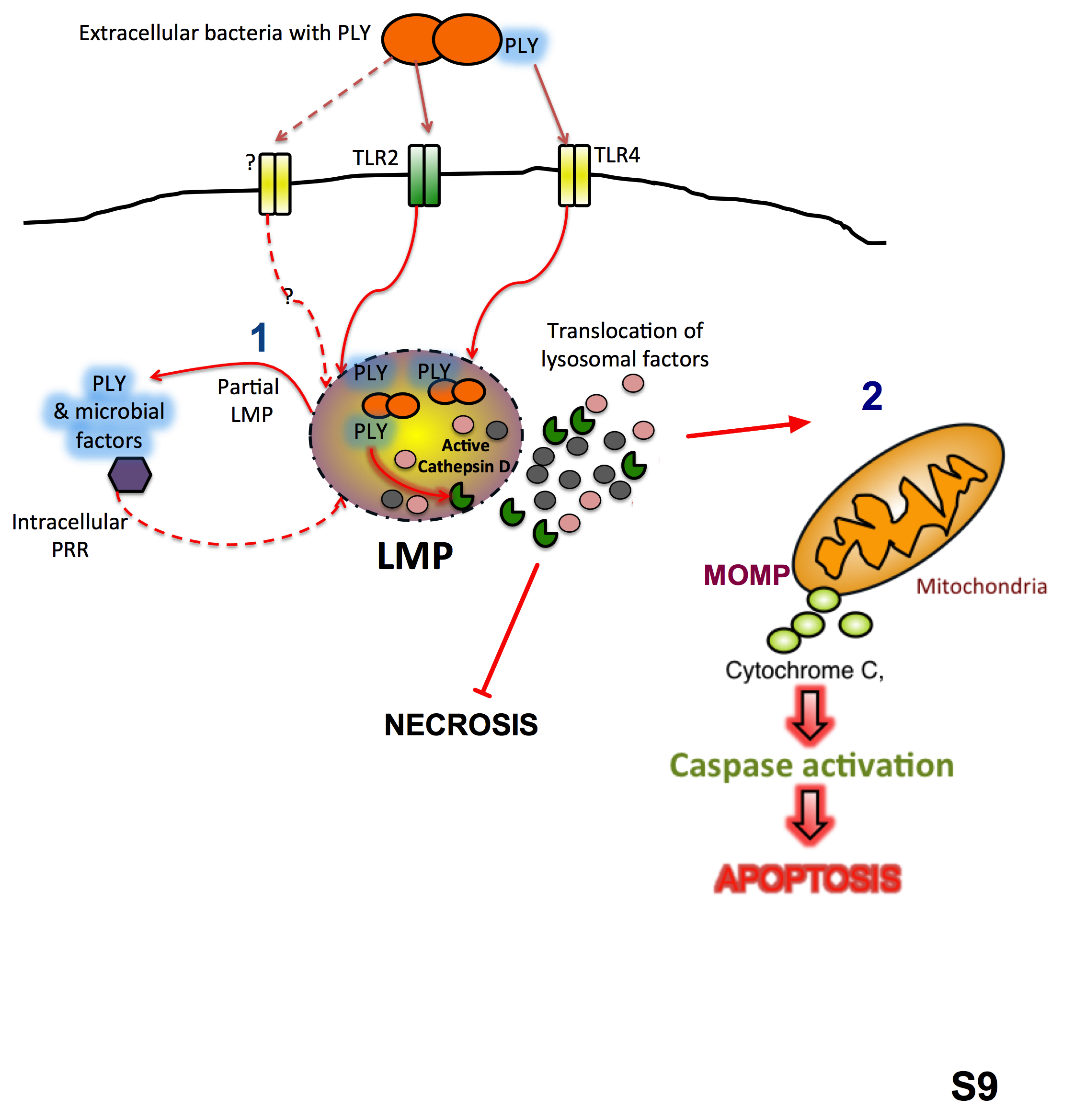

Supplement: Figure S9 — Model of lysosomal membrane permeabilization in S. pneumoniae-challenged macrophages. Pneumolysin (PLY) acts in two stages to stimulate apoptosis. In the first stage (1), the early phase of macrophage sensitization for apoptosis involves PLY, which, acting independently of its pore-forming capacity but in concert with other microbial factors, induces lysosomal/phagolysosomal membrane permeabilization (LMP), in a process that is independent of bacterial phagocytosis. This process involves Toll-like receptor 2 (TLR2) and TLR4 and potentially other signals generated through additional pattern recognition receptors (PRRs). Intracellular bacteria release PLY during LMP, which progressively accumulates in the cytosol, where it has the potential to engage additional unidentified pathways. Intracellular PLY is required for maximal activation of cathepsin D and for engagement of the second phase of apoptosis (2) through execution of the apoptosis program downstream of LMP. This involves mitochondrial outer membrane permeabilization, activation of caspase 9 and 3, and, ultimately, nuclear fragmentation, which ensures the death of the macrophage by apoptosis rather than necrosis. Phagocytosis-independent PLY signals induce LMP but are also associated with cytokine generation, while the phagocytosis-dependent responses induced by PLY that are associated with apoptosis induction are also associated with other aspects of antimicrobial host defense such as nitric oxide generation. Download [file mbo005142021sf9.tif]
